# The Climate–Migration–Health Nexus: A Multisectoral Framework for Action, with Case Insights from MENA

**DOI:** 10.3390/tropicalmed11030079

**Published:** 2026-03-11

**Authors:** Davide T. Mosca, Michela Martini

**Affiliations:** 1 Realizing Health SDGs for Migrants, Displaced, and Communities, Nairobi P.O. Box 3376-00200, Kenya; 2 Regional Office for the Middle East and North Africa (MENA), International Organization for Migration (IOM), Cairo 11211, Egypt

**Keywords:** climate–migration–health nexus, climate-resilient health systems, migration health, health equity, MENA region

## Abstract

The convergence of climate change, migration, and health represents a critical global challenge, with the Middle East and North Africa (MENA) region illustrating acute vulnerabilities while offering insight relevant beyond the region. Increasing exposure to extreme heat, droughts, and floods drives displacement, constrained mobility, and adaptive migration, placing additional pressure on already stretched health systems. This paper proposes an integrated *Nexus Action Framework for Climate Change, Migration, and Health* (NAF-CMH) to address these interlinked dynamics and move beyond fragmented, sector-specific responses. The framework conceptualizes human mobility both as a potential resilience strategy and as a determinant of health, encompassing climate-affected migrants, displaced populations, and those experiencing involuntary immobility across diverse pathways and settings. It promotes systematic integration of health considerations into climate adaptation and migration governance and situates these interventions within the broader agenda of *climate-resilient health systems*. Drawing on a non-systematic narrative review of peer-reviewed and grey literature, complemented by the authors’ expertise, the paper identifies seven interrelated pillars for coordinated policy and operational action. While grounded in MENA-specific vulnerabilities, the framework is flexible and adaptable to other regions facing climate-driven mobility challenges. By providing an operational architecture for multisector collaboration, the NAF-CMH supports policymakers, public health authorities, and migration actors in strengthening resilience, reducing vulnerability and safeguarding health amid accelerating climate impacts and evolving mobility patterns.

## 1. Introduction

The Middle East and North Africa (MENA) region—as operationally defined in this paper for analytical and programming purposes, drawing on the International Organization for Migration (IOM) regional delineation (For IOM and the purpose of this paper, the MENA region encompasses 18 countries and territories: Algeria, Bahrain, Egypt, Iran, Iraq, Jordan, Kuwait, Lebanon, Libya, Morocco, Oman, Qatar, Saudi Arabia, Sudan, Syrian Arab Republic, Tunisia, United Arab Emirates, Yemen, and the Occupied Palestinian Territories. While regional classifications vary across institutions, the use of the IOM definition in this paper is intended to ensure analytical consistency rather than to imply a fixed or normative geopolitical boundary. Importantly, the proposed framework and its insights are not contingent on this specific regional delineation and are applicable to other contexts experiencing comparable climate-, migration-, and health-related dynamics)—is characterized by a convergence of protracted crises, socio-economic inequalities, political instability, and acute environmental pressures, compounded by a high vulnerability to climate change and climate-related hazards (WB 2021) [1].

As one of the world’s most climate-affected regions globally, MENA is experiencing rising temperatures, desertification, water scarcity, extreme weather events, ecosystem degradation, and deteriorating air quality. At the same time, exposure, adaptive capacity, and resource availability vary widely across the region, shaping uneven abilities among countries and communities to anticipate, absorb, and respond to climate-related risks.

Climate and environmental stressors increasingly undermine livelihoods and key economic sectors in the MENA region, including agriculture, construction, and formal and informal labour, reducing productivity and income security (WB 2023) [2]. These pressures interact with demographic trends and structural inequalities to generate diverse migration and mobility patterns, including internal displacement, cross-border movements, and labour migration. Much of this mobility occurs within the region, often toward urban or economically active areas that are themselves exposed to environmental stress and climate hazards (IOM, 2023) [3].

Terminology and definition related to migration and displacement vary across legal, policy, and academic domains, shaping how populations, exposures, and vulnerabilities are characterized and addressed. In line with international policy frameworks and current international practice, this paper uses the term “migration” as an umbrella concept encompassing human mobility in all its forms, including voluntary and forced migration, internal displacement, cross-border movements, and involuntary immobility. (*Migration*—The movement of persons away from their place of usual residence, either across an international border or within a State; *Climate Migration*—The movement of a person or group of persons who predominantly for reason of sudden or progressive change in the environment due to climate change, are obliged to leave their habitual place of residence, or chose to do so, either temporarily or permanently, within a Sate or across an international border. Source: IOM Key Migration Terms. https://www.iom.int/key-migration-terms accessed on 12 October 2025). This definitional choice is not merely semantic; it conditions how risk profiles are constructed, which populations are rendered visible in policy responses, and how vulnerability and resilience are assessed within climate-affected contexts.

These dynamics have significant and multi-layered implications for health. Climate change exerts direct effects on health through increased exposure to heat stress, air pollution, water scarcity, food insecurity, and climate-sensitive diseases (WHO, 2023 [4]; Borghesi S. and Ticci E., 2019 [5]). These impacts occur independently of mobility and progressively strain health and social systems, particularly in contexts of pre-existing vulnerability and system fragility. At the same time, climate stressors—including extreme weather events, slow-onset environmental degradation, and livelihood disruption—affect human mobility, influencing who moves, where, and under what conditions. Displacement, unplanned urbanization, constrained migration or relocation, and involuntary immobility often represent adaptive, risk-amplifying, or structurally constrained responses to environmental pressures. These mobility dynamics do not merely respond to risks; they reshape them by altering where people live, work and access services, modifying exposure profiles (e.g., environmental hazards, vector ecology, overcrowding), continuity of care, legal protection, and social network. Mobility thus functions both as a determinant and modifier of health risk, influencing the distribution of vulnerability and resilience across populations and over time.

Climate-related migration can exacerbate inequalities and social tensions, particularly in settings with inadequate infrastructure and governance (IOM 2024) [6], Under these conditions, mobility dynamics may intensify competition over resources, strain public services, and heighten both physical and mental health vulnerabilities. Migration thus functions not only as a response to environmental stress but also as a determinant of risk, with outcomes shaped by the broader social, economic, and institutional context. Health system capacity and governance responses mediate these interactions. Preparedness, financing, policy coherence, and intersectoral coordination determine whether climate-related mobility amplifies vulnerability—through service disruption, exclusion, and cumulative risk—or contributes to adaptive resilience. Over time, these interactions generate dynamic feedback loops: deteriorating health and weakened systems can increase susceptibility to future climate shocks and trigger further displacement or precarious mobility, while inclusive urban planning, migrant-sensitive health policies, and strengthened, climate-resilient primary care systems can buffer risks, reduce inequality, and enhance collective adaptive capacity across the climate–mobility–health nexus.

Within this context, migrants, displaced populations, and other marginalized groups face disproportionate vulnerabilities, with women and girls among them particularly affected, experiencing persistent barriers to accessing healthcare, reproductive health risks, and heightened exposure to gender-based violence (WB, 2021) [1]. For many individuals and households, migration emerges as a spontaneous or adaptive response to climate and environmental pressures. However, in the absence of supportive policies and favorable societal conditions, migration can exacerbate vulnerabilities (Mosca et al., 2020) [7], particularly when individuals relocate to areas already exposed to climate-related hazards.

The severity of these vulnerabilities is shaped by the interaction between the intensity of climate hazards, levels of exposure, and the capacity of individuals and communities to build resilience through adaptive strategies. Migrants and displaced populations often remain marginal to adaptation processes, leaving them disproportionately exposed as climate-related risks intensify (IPCC 2023) [8]. Evidence, integrating climate change, migration, and health remain limited, particularly in the MENA region, highlighting the need to combine regional examples with global evidence to inform effective policy and operational guidance. In the MENA region, climate-related pressures frequently intersect with protracted conflict, political instability, economic fragility, and large-scale displacement dynamics. The interaction of these environmental and structural drivers shapes mobility patterns and amplifies health and social vulnerabilities, making it difficult to isolate environmental stressors from broader contextual conditions. Consistent with this, available evidence indicates that vulnerability in MENA contexts is determined by the combined effects of environmental exposure, governance capacity, and conflict-related displacement.

At the same time, mobility is not equally accessible to all. Structural, economic, legal, and security-related constraints restrict the capacity to move for many populations, resulting in conditions of involuntary immobility and prolonged exposure to climate- and conflict-related risks. The concept of in/mobility—encompassing both the capacity and the inability to move (Schewel, 2020) [9]—is therefore central to understanding adaptation dynamics in fragile and climate-affected settings. Addressing these interconnected challenges requires an integrated approach that recognizes mobility as both a potential adaptation strategy and a source of risk, while prioritizing equity, resilience, and health protection in areas under climate pressure (McMichael 2020) [10].

A growing body of international policy instruments now recognises the importance of safeguarding the health and well-being of migrants and displaced populations across climate action, migration governance, and public health agendas. Yet fragmented governance structures, sectoral silos, and limited operational guidance continue to hinder the translation of these commitments into inclusive action, highlighting the need for a coordinated approach across the climate–migration–health nexus, as migrants and displaced populations remain insufficiently prioritised in adaptation, resilience planning, and health system responses (Silenzi et al. 2023) [11].

The rationale for adopting an integrated, action-oriented climate–migration–health framework to address these governance and implementation gaps is outlined in Box 1.

Box 1 **Why an integrated, action-oriented climate–migration–health framework is needed.**

 An integrated climate–migration–health action framework can support more coherent, people-centred public health responses to climate-related mobility by addressing the limitations of fragmented, sector-specific approaches. Its added value may be seen in the following areas:

1. *Accounting for lived mobility and exposure patterns*. Climate change affects health through both direct exposures and mobility pathways—including displacement, labour migration, planned relocation, and involuntary immobility—shaping risks across origin, transit, destination, and return contexts.
2. *Enabling prevention and anticipatory action*. Linking climate hazards, mobility dynamics, and health outcomes can strengthen early warning, risk anticipation, and preventive public health planning.
3. *Re-centering health in climate and migration responses*. The framework highlights health as both an outcome and a determinant of climate mobility, encouraging rights-based and inclusive approaches for migrants, displaced populations, and host communities.
4. *Strengthening policy coherence and preparedness*. Greater alignment across climate adaptation, migration governance, and public health preparedness can enhance surveillance, continuity of care, and emergency response capacities.
5. *Supporting context-specific, evidence-informed analysis*. An integrated lens facilitates the identification of differentiated health risks and vulnerabilities along mobility pathways, including in urban, border, fragile, and crisis-affected settings.
6. *Advancing equity and resilience*. By making structural and social inequities more visible, the framework can inform more targeted interventions and contribute to strengthening the resilience of individuals, communities, and health systems.
7. *Providing a shared reference point*. A common analytical framework can facilitate coordination across climate, health, migration, and development actors, supporting dialogue, joint programming, and the translation of global commitments into context-specific strategies.

Based on an integrative, policy-oriented review of selected narrative literature, this paper examines the Climate–Migration–Health (CMH) nexus with the aim of identifying actionable policy and operational components for an integrated response. It presents the core elements of a proposed *Nexus Action Framework for Climate, Migration, and Health* (NAF-CMH), drawing on challenges highlighted in the literature as well as the authors’ professional experience. While grounded in the specific vulnerabilities of the MENA region, the framework is intentionally flexible and adaptable to other contexts affected by climate change and human mobility. It emphasizes the active participation and agency of affected communities in shaping interventions.

### 1.1. Conceptual Positioning of the Nexus Action Framework

Existing *climate-resilient health system* models—particularly the 2015 WHO framework [12]—have provided an essential foundation by integrating climate considerations across the six health system building blocks. Migration Health frameworks have similarly advanced understanding of equity, access, and service delivery for mobile populations. However, these approaches have mainly evolved within parallel policy and institutional spaces. Climate-resilient health models have not systematically incorporated human mobility as a structural determinant of system functioning, while migration health frameworks have only recently begun to engage more explicitly with climate change as a driver of health system transformation. Important efforts led by the World Health Organization—including the operationalization of the Global Action Plan on Refugee and Migrant Health—represent significant steps toward convergence.

Nevertheless, despite growing recognition and emerging collaboration across domains, the intersections between climate change, human mobility, and health systems remain insufficiently structured within unified operational architectures. As a result, implementation continues to be shaped by sector-specific mandates, funding streams, and governance arrangements, limiting the development of fully integrated responses.

This narrative review employs an iterative and interpretive synthesis of peer-reviewed and grey literature to examine these domains as structurally interconnected rather than sequential or sectoral. Drawing on established policy platforms—including climate-resilient health systems, the migration health agenda, and safe, orderly, and regular migration—the analysis identified recurring themes and areas of convergence across the literature. These convergences were progressively consolidated into seven interrelated pillars, which do not introduce new thematic domains but rather reframe cross-sector established priorities within a single system-oriented and multisector architecture that explicitly treats climate change and human mobility as interacting structural forces shaping health governance.

The paper is organized around three interrelated policy dimensions essential to operationalizing the CMH nexus:

1. *Global commitments and policy alignment*, positioning the NAF-CMH within key international frameworks—including the Paris Agreement and UNFCCC processes, the Global Compact for Migration (GCM), the Global Compact on Refugees (GCR), the Sendai Framework for Disaster Risk Reduction, and the Sustainable Development Goals (SDGs)—to promote coherent and mutually reinforcing action.
2. *Health system resilience and strengthening*, building on WHO guidance, to address climate-related health risks and service gaps affecting migrants, displaced populations, and host communities, while integrating local knowledge, participation, and culturally responsive approaches to enhance adaptive capacity and long-term system performance.
3. *Partnerships and governance*, advancing cross-sectoral, interagency, and whole-of-government approaches to enable coordinated planning, advocacy, and resource mobilization across humanitarian, development, and peace actors, while embedding community participation and affected populations’ perspectives in decision-making.

The proposed NAF-CMH advances the field by offering a systems-oriented framework that brings climate governance, migration governance, and health system strengthening into a shared analytical space. It aligns health systems thinking with broader global policy agendas—including climate action, development frameworks, and migration platforms—illustrating how sector-specific and cross-sector initiatives intersect within a common Climate–Human Mobility–Health perspective. Rather than merging institutional mandates, the framework clarifies relationships, identifies areas of convergence, and aims to structure dialogue across domains that have often evolved in parallel.

A central feature of the NAF-CMH is its integration of structural determinants within its conceptual architecture. Political economy dynamics, labour market conditions, regulatory frameworks, and migration governance regimes are considered cross-cutting factors shaping mobility patterns and health system responses. These determinants influence who moves, under what circumstances, and with what access to services and social protection.

By situating these factors within its governance and policy dimensions, the NAF-CMH provides an analytical lens for examining how structural and institutional conditions shape the interactions between climate change, human mobility, and health systems. In doing so, the framework treats these factors not as background context, but as integral elements of the Climate–Human Mobility–Health nexus.

While the framework is anchored in the MENA region for contextual illustration, examples from these countries are presented as illustrative rather than comprehensive validation. Global evidence and operational frameworks were used to supplement regional data gaps and support conceptual transferability to other climate- and mobility-affected settings.

### 1.2. Methodology

*Review Design and Scope*. This paper employed a structured, non-systematic narrative review to examine the Climate–Migration–Health (CMH) nexus at the systems and policy level, with the primary objective of informing the conceptualization of a policy-oriented Nexus Action Framework (NAF-CMH). The review focused on the Middle East and North Africa (MENA) region as a contextual anchor, while also drawing on global literature where MENA-specific integrative studies are limited. The review period covers 2016–2025, reflecting the timeframe following key global policy milestones, including the 2030 *Agenda for Sustainable Development*, the *Paris Agreement*, and the *New York Declaration on Refugees and Migrants*.

*Scope of Evidence and Chapter Use*. The structured narrative review informed:

Chapter 3—“Mapping the Climate-Migration-Health Nexus: Concepts and Emerging Evidence”: Synthesizing peer-reviewed, conceptual, and policy-relevant studies to identify recurring themes and patterns across climate, migration, and health domains.
Chapter 4—“From Evidence to Action: Framing a Nexus Action Framework for Climate, Migration, and Health”: Translating the synthesis into actionable framework components for governance, health systems, and policy integration.

While the core analysis is based on a structured, non-systematic narrative review, Chapter 2 “Regional Context for Action: Climate Risks, Human Mobility, and Health Challenges in MENA” relies primarily on complementary secondary sources—including institutional reports, country assessments, and additional literature—that were not part of the formal review corpus. This distinction clarifies that contextual description of the MENA region draws on broader references, whereas the review methodology described below informs the evidence synthesis and framework development in Chapters 3 and 4.

*Search Strategy.* A staged search strategy was implemented to ensure conceptual breadth while maintaining relevance to systems and governance dimensions of the CMH nexus.

1. *Database Searches*

Structured searches were conducted in PubMed and Google Scholar using the Boolean search logic presented in Box 2.

Box 2 **Search terms and Boolean logic used in the literature review.**

((“climate change” [Title/Abstract] OR “climate adaptation” [Title/Abstract] OR “environmental change” [Title/Abstract]) AND (migration[Title/Abstract] OR displacement[Title/Abstract] OR “human mobility” [Title/Abstract] OR refugees[Title/Abstract]) AND (“health system*” [Title/Abstract] OR “health policy” [Title/Abstract] OR governance[Title/Abstract] OR “service delivery” [Title/Abstract] OR “system resilience” [Title/Abstract]))

Country-specific terms (e.g., Morocco, Lebanon, Jordan, Egypt) were initially added to refine results for MENA contexts. These searches yielded not fully integrative CMH studies focused on MENA. Removing the country-specific restriction and focusing on the CMH nexus globally produced 33 potentially relevant publications, including peer-reviewed studies, editorials, and position papers in specialized journals. Following title and abstract screening for relevance to governance or health system dimensions across all three CMH domains, a subset of these documents was retained for further analysis.

2. *Grey Literature and Institutional Sources*

Given the policy and operationally oriented nature of the CMH nexus, targeted grey literature was included from multilateral and regional organizations (e.g., WHO, IOM, UNHCR, IPCC, World Bank). Inclusion was restricted to sources that:

- Addressed governance, systems-level planning, or institutional coordination.
- Provided operational frameworks or programmatic evaluations.
- Contributed conceptual clarity to nexus integration.

Purely descriptive reports without governance or systems-level relevance were excluded. Peer-reviewed literature formed the primary conceptual base, while the grey literature supplemented operational and governance perspectives not yet extensively represented in academic research or provided theoretical context.

3. *Screening and Selection Process*

Screening was conducted in sequential stages. Titles and abstracts were reviewed for relevance to all three CMH domains and for engagement with governance, policy, or systems-level considerations. Articles meeting these criteria underwent full-text assessment. An iterative reassessment process ensured alignment with inclusion criteria as conceptual themes evolved during synthesis. Backward and forward citation tracking (snowballing) was used to identify additional relevant sources. Selection decisions prioritized integrative analyses over narrowly sectoral studies. Sources were excluded if they:

- Did not address all three CMH domains.
- Focused exclusively on clinical or epidemiological outcomes without governance implications.
- Presented localized adaptation projects without systemic relevance.

The combination of initial database searches, targeted grey literature, and snowballing resulted in 39 core sources retained for qualitative synthesis, supplemented by additional contextual materials.

*Inclusion and Exclusion Criteria.* The following inclusion criteria were applied:

- Published in English between 2016 and 2025.
- Addressed all three CMH domains (with preference for integrative analyses).
- Engaged at governance, policy, or systems level.
- MENA-focused or globally applicable to fragile or mobility-affected contexts.

The following exclusion criteria were applied:

- Purely biomedical or epidemiological studies.
- Studies lacking governance/systems relevance.
- Non-analytical commentary without substantive framework contribution.

*Quality Considerations*. Given the narrative, policy-oriented design, no formal risk-of-bias scoring tool was applied. Methodological rigor was ensured through structured appraisal of each source’s conceptual integration across climate, migration, and health domains; explicit engagement with governance or systems-level dimensions; and analytical coherence. Peer-reviewed publications were prioritized for analytical grounding, while institutional and grey literature provided operational and policy perspectives. Sources lacking substantive analytical contribution were not retained.
*Data Extraction and Synthesis*. Data extraction focused on identifying:

- Governance mechanisms.
- Health system adaptation strategies.
- Cross-sector coordination mechanisms.
- Population vulnerability considerations.

Synthesis was thematic rather than quantitative. Key governance and systems-level priorities were identified and mapped across academic and institutional sources. The seven pillars of the NAF-CMH were derived through an iterative thematic synthesis of the literature, consolidating recurring priorities across climate–health, migration governance, and health system resilience frameworks. These pillars do not introduce new domains but organize and operationalize priorities already present in the literature. Thematic saturation was understood conceptually, as the point at which additional sources did not provide substantially new insights relevant to governance or systems-level considerations.

*Regional Contextualization (MENA).* Although anchored in MENA, the limited availability of integrative CMH studies in the region required careful distinction between:

- Findings supported by MENA-specific evidence.
- Conceptual extrapolations derived from global literature.
- Policy analogues applied to MENA contexts.

Illustrative examples from MENA were included for each NAF pillar where available, serving as contextual anchors rather than comprehensive regional validation.

*Limitations and Uncertainty.* This paper is based on a non-systematic, narrative review and should be interpreted as a policy-oriented synthesis rather than an empirically validated causal analysis. Several factors shape the evidence base and interpretation of findings:

- *Attribution Complexity*—Migration decisions are multi-causal, complicating direct attribution to climate drivers.
- *Conceptual and Definitional Variability*—Terms such as “migrant,” “refugee,” “migration,” “climate-related mobility” differ across legal, policy, and academic contexts. This review applies consistent functional definitions aligned with international frameworks while acknowledging ongoing conceptual and normative debates.
- *Regional Evidence Gaps*—Integrative peer-reviewed CMH research in the MENA region remains limited, constraining local empirical depth.
- *Heterogeneity of Sources*—Grey and multilateral literature captures operational and governance perspectives but varies in format and methodological reporting standards.
- *Data Uncertainty and Measurement Constraints*—Migration and displacement data, particularly in conflict-affected and climate-exposed regions, are characterized by definitional inconsistencies, underreporting of internal and irregular mobility, and variability across institutional data systems. Political sensitivities and fragmented reporting mechanisms further limit comparability and precision. As a result, the evidence base should be interpreted as indicative of systemic patterns rather than definitive quantitative attribution.

Given these considerations, the findings are indicative rather than comprehensive. They highlight priority areas for further empirical research, including comparative country studies, locally documented case studies, and applied field-based evaluations to refine and adapt the NAF-CMH in context-specific ways.

## 2. Regional Context for Action: Climate Risks, Human Mobility, and Health Challenges in MENA

Based on United Nations and national population estimates 2024, the IOM-defined Middle East and North Africa (MENA) region comprises approximately 465 million inhabitants, including an estimated 44 million international migrants and refugees (around 9.5% of the total population), with figures varying depending on regional delineation and data availability. This represents one of the largest regional migrant populations globally and underscores the MENA region’s role as a major origin, transit, and destination hub.

Migration and human mobility are central to livelihoods across the region and involve complex patterns of regular and irregular movement, including intra-regional flows and onward migration toward North Africa, Europe, and the Gulf. These movements are shaped by inter-country economic disparities, high youth unemployment, conflict, environmental stressors, and health risks, including gendered and structural drivers that influence vulnerability and mobility pathways (Diab, and Scissa, 2023) [13]. In the MENA region, climate-related pressures intersect with protracted conflict, political instability, and economic fragility, while political economy dynamics, governance arrangements, and institutional capacity collectively shape adaptation and health responses (Diab 2024) [14]. Migration decisions typically reflect multiple, concurrent factors, with climate change acting as either a primary or secondary driver (IOM 2025) [15]. This underscores that environmental stressors cannot be considered in isolation from broader structural and conflict-related conditions, which together shape mobility, vulnerability, and adaptive capacity across the region (Zaccara et al. 2021) [16].

Mobility dynamics in the MENA region broadly fall into three interrelated patterns: labour migration, mixed migration, and displacement; (*Mixed migration* or *mixed movements* refers to movements in which a number of people are travelling together, generally in an irregular manner, using the same routes and means of transport, but for different reasons. People travelling. as part of mixed movements have varying needs and profiles and may include asylum seekers, refugees, trafficked persons, unaccompanied/separated children, and migrants in an irregular situation) (IOM, 2025) [15]. While this analysis adopts the IOM regional definition for analytical coherence, it recognizes substantial variation in how these mobility patterns manifest across countries, shaped by differences in income levels, governance capacities, and health system resilience. Consequently, the Nexus Action Framework is presented as a flexible, non-prescriptive tool, designed for adaptation to diverse sub-regional contexts—including labour-migration–dominated settings in high-income *Gulf Cooperation Council* (GCC) countries, mixed migration, transit contexts, and displacement-driven situations in states facing conflict, economic fragility, and climate-related hazards.

Labour migration is a cornerstone of economic and social development in the MENA region, sustaining key productive sectors and generating substantial remittance flows to countries of origin. Remittances represent a significant share of national income in several MENA countries—averaging approximately 17% of GDP in Jordan, 14% in Lebanon, and 7% in Morocco between 1990 and 2018—highlighting the macroeconomic importance of migrant labour (Miniaoui et al., 2019) [17]. These contributions, however, are highly sensitive to health and employment shocks. The COVID-19 pandemic illustrated this vulnerability, as illness, mobility restrictions, and job losses disrupted labour supply and remittance flows, with cascading effects on households, health systems, and local economies, emphasizing that migrant health is a key determinant of regional economic resilience.

Gulf countries are major destinations for labour migration, with migrants comprising nearly half of the workforce in Arab states—the highest proportion globally (ILO, 2023) [18]. Many of these workers are low-skilled and concentrated in sectors such as construction, hospitality, and domestic work, where poor working conditions and health risks are common, and increasingly exacerbated by climate-related stressors.

More than a third of migrants in the MENA region are forcibly displaced across borders, making the region the largest source of refugees globally. Prolonged conflicts, natural hazards, and food insecurity have also driven extensive internal displacement, with over 22.2 million internally displaced persons (IDPs) recorded in 2022 (IOM/Global Data Institute 2023) [19], placing MENA second worldwide only to sub-Saharan Africa. Displacement and migration patterns are increasingly shaped by anthropogenic climate change. Following terminology widely used in the Intergovernmental Panel on Climate Change (IPCC) literature, this refers to long-term changes in climate patterns primarily driven by human activities—particularly greenhouse gas emissions and land-use changes—rather than by natural climate variability. These climate pressures interact with demographic, economic, political, social, and environmental factors, often amplifying risks and vulnerabilities for affected populations. While conflict remains the primary driver of displacement (IOM/Global Data Institute, 2023) [19], climate-related hazards are emerging as significant contributors to both internal and cross-border displacement (IDMC 2022) [20] and to voluntary migration, with impacts projected to intensify in the coming decades (Beyer et al., 2023) [21]; Cárdenas-Vélez et al., 2024) [22]. As a threat multiplier, climate change exacerbates existing vulnerabilities, underscoring the need for integrated adaptation and resilience strategies that address human mobility and health in tandem (FH Abdullah 2023) [23].

Countries in the MENA region are highly exposed to climate hazards, including extreme heat, severe water scarcity, low rainfall, and declining arable land, compounded by rapid urbanization and uneven adaptive capacities (IOM 2023) [24]. Key climate-related challenges affecting the MENA region are summarized in Box 3. As the world’s most water-stressed region, home to 14 of the 25 most water-scarce countries globally, MENA is projected to experience rising temperatures and accelerated drying trends that exceed the global average, with significant consequences for human and animal health as well as economic and social stability.

Box 3 **Key Climate issues in the MENA.**

➢ Between 1980 and 2022, temperatures in the MENA region rose by 0.46 °C per decade, well above the global average of 0.18 °C (Boehm S, Schumer C., 2023) [25]. The IPCC projects a further 2 °C rise within the next 15–20 years, alongside reduced precipitation, resulting in a 20–30% decrease in water runoff by 2050. These changes will worsen water scarcity in countries like Iraq, Sudan, and Yemen, and increase drought risks in Jordan, Iraq, and Syria. Recent droughts in Morocco in 2022 and in Tunisia in 2023 underscore the urgency. Urban centers with better water access, such as Algiers, Cairo, and Tunis, are likely to become climate migration hotspots (IOM 2023) [3].
➢ Higher temperatures increase evaporation, depleting water resources and affecting agriculture and ecosystems. Sudden heavy rains after dry periods can cause surface runoff and flooding, as the soil struggles to absorb excess moisture. In Sudan, the 2020 flooding worsened an ongoing humanitarian crisis, damaging infrastructure and spreading waterborne diseases like cholera. Flooding in Yemen in 2022 and 2024) exacerbated displacement, while in Iraq, floods from 2016 to 2022 displaced over half of the affected families. In Syria, the 2023 floods destroyed tents in displacement camps, worsening the crisis. Major floods in Libya in 2019 and, 2023 caused significant loss of life and livelihoods and led to large-scale displacements (IOM 2023) [3].

Recurring climate-related hazards underscore the MENA region’s exposure to the compounded effects of climate change on human mobility and health. Sudden-onset events—such as floods and storms—frequently trigger acute internal displacement by posing immediate threats to life and destroying housing, land, and essential services (IOM, 2023) [3]. In contrast, slow-onset processes, including desertification and sea-level rise, progressively erode habitability and livelihoods, shaping longer-term and often incremental migration decisions.

When climate-related mobility unfolds in contexts already characterized by environmental stress, resource constraints, and limited service-capacity, vulnerabilities may deepen—particularly where health needs are not systematically anticipated or addressed. Climate shocks and population inflow can undermine incomes, food security, and basic services, intensifying pressure on housing, water, employment, and healthcare systems. These cumulative stresses may strain infrastructure and social cohesion, heighten tensions between mobile populations and host communities, and, in some settings, contribute to instability or conflict (Henderson et al., 2014) [26]; (IOM, 2023) [27]; (Kim K., Ferré Garcia T. 2023) [28]. The interaction between climate change and human mobility thus generates a distinct and evolving profile of health risks. These include undernutrition; climate-sensitive infectious diseases; mental health conditions linked to displacement, uncertainty, and loss; stigma and marginalisation; and barriers to accessing essential services. Such risks frequently compound pre-existing socio-economic and health system constraints, limiting coping capacity and affecting both mobile populations and host communities. Together, these dynamics underscore the need for integrated responses that address climate change, human mobility, and health as interconnected determinants of resilience and social cohesion at both community and systems levels (UNHCR 2024) [29]. Table 1 provides an overview of major climate- and mobility-related drivers, associated health risks, the populations and systems most affected, and selected case examples from the MENA region and beyond.

Beyond the direct health impacts of climate change, human mobility across the MENA region reveals marked disparities in health system capacity and preparedness. Countries differ substantially in their ability to prevent, absorb, and respond to health risks associated with climate stressors and population movements. While some benefit from relative political stability, stronger institutions, and greater financial resources, others face compounded constraints linked to conflict, weak governance, limited fiscal space, and high climate vulnerability, with ongoing and recent conflicts in parts of the region further aggravating displacement pressures and health system strain. These structural differences translate into wide variation in health system preparedness, from comparatively well-developed services in countries such as Tunisia and Morocco to fragile and underfunded systems in conflict-affected settings, including Syria and Yemen, as well as contexts under severe socio-economic and political stress, such as Lebanon.

Country experiences illustrate uneven progress across the region. Qatar has introduced protections for outdoor migrant workers exposed to extreme heat, demonstrating the value of targeted climate-related occupational health measures (Al Thani 2023) [50]. Jordan has advanced the integration of refugee health services into national systems, while Lebanon’s protracted economic crisis has weakened health system capacity and preparedness for climate-related threats. Although progress toward *Universal Health Coverage* (UHC) has been made in parts of MENA, equitable access to essential services remains uneven (Alshehari et al., 2024) [51] and climate shocks risk widening these gaps. Recognizing human mobility as a core dimension of climate change strengthens the case for climate-resilient primary health care aligned with UHC principles. Importantly, addressing climate-related mobility creates opportunities to advance health outcomes through coordinated investments across multiple domains—including labour protections, social protection systems, urban planning, water and food security, and disaster risk reduction. Aligning climate adaptation financing with inclusive health system strengthening can therefore enhance service delivery, financial protection, and resilience for both mobile and host communities, advancing equity and long-term system sustainability.

Against a backdrop of intensifying climate risks, diverse mobility dynamics, and uneven development trajectories across the MENA region, sectoral approaches may be insufficient to fully address the interconnected health and social impacts that emerge. A growing body of scholarship highlights the importance of integrated and multidisciplinary approaches to research, programming, and policy in such complex contexts (Lasater et al. 2025) [52]. Addressing these challenges requires analytical attention to how strategic investments in primary healthcare, livelihoods, social protection, and essential services intersect within migrant-inclusive local systems. Certain interventions may need to be situated within the humanitarian–development–peace (HDP) continuum, linking immediate responses with longer-term resilience-building in contexts affected by conflict, fragility, and climate stressors (Mena et al., 2023) [53]. Meanwhile, broader health and mobility issues span development and governance domains. From this perspective, there is a need to systematically consider structural vulnerabilities, cross-sector interdependencies, and contextual heterogeneity across the region. Integrated approaches that connect climate, mobility, and health concerns can help clarify how risks intersect with broader development and governance agendas—including the *Sustainable Development Goals* (SDGs) and the *Global Compact for Migration* (GCM)—and support efforts to strengthen resilience, equity, and system-level preparedness in fragile, conflict-affected, and climate-stressed settings.

## 3. Mapping the Climate–Migration–Health Nexus: Concepts and Emerging Evidence

This chapter draws on a non-systematic narrative review of selected literature to identify key policy, governance, and operational components relevant to the development of an action-oriented framework at the intersection of climate change, migration, and health.

While the importance of climate–migration–health linkages has gained increasing recognition within global climate and health agendas, the literature suggests that these connections remain unevenly articulated and insufficiently translated into coherent, coordinated multisectoral action. In particular, the integration of migrant health into climate policy and migration debates remains fragmented, highlighting the need for more structured, action-oriented approaches that can translate growing awareness and knowledge into aligned policy and operational responses across sectors. Box 4 provides background on the evolution of the global climate policy architecture. Within this evolving policy landscape, health has gained increasing visibility in climate-related discourse, including through IPCC assessments and the expanding engagement of the World Health Organization (WHO).

Box 4 **Evolution of the Global Climate Policy Architecture Relevant to Climate, Migration, and Health.**

The growing prominence of environmental and climate change concerns on the global agenda over recent decades has significantly shaped the policy frameworks within which climate, migration, and health are addressed (Jackson 2007) [54]. Early international attention emerged with the 1972 *Stockholm Conference* [55], which framed environmental degradation and climate-related risks as global concerns and called on governments to assess and mitigate human-induced environmental harm.

This momentum was consolidated with the establishment of the *Intergovernmental Panel on Climate Change* (IPCC) in 1988 (WMO/UNEP, 1988) [56], tasked with assessing scientific evidence on climate change, greenhouse gas emissions, and their impacts on ecosystems, societies, and human well-being.

A major governance milestone followed with the adoption of the *United Nations Framework Convention on Climate Change* (UNFCCC) [57] at the 1992 *Rio Earth Summit*. The UNFCCC established shared principles, commitments, and institutional mechanisms—most notably the Conference of the Parties (COP)—to guide global climate action. It emphasized climate monitoring, emissions reduction, scientific cooperation, and the mitigation of environmental, economic, and public health consequences of climate change.

The *Fourth IPCC Assessment Report* in 2007 warned that climate change was worsening disease burdens and premature deaths, particularly in vulnerable communities, undermining public health progress (Adger N. et al. AR4, 2007) [58]. In response, the 2008 World Health Assembly Resolution on *Climate Change and Health* (WHA61.19) [59] urged WHO to engage with the UNFCCC. That same year, Resolution WHA61.17 [60] on the *Health of Migrants* called for migrant-sensitive health policies and equitable healthcare access, but did not explicitly address climate-induced migration, reflecting ongoing challenges in linking these issues.

The 2009 *Lancet*–UCL Commission on *Managing the Health Effects of Climate Change* (Costello A. et al. 2009) [61] identified climate change as the greatest global health threat of the 21st century. Building on findings of the 2008 WHO Commission on *Social Determinants of Health*, the Commission demonstrated how climate change exacerbates existing social and structural vulnerabilities, with disproportionate impacts on marginalized populations. Human mobility—including displacement and migration—was recognised as a critical pathway through which climate change translates into adverse health outcomes, alongside changing disease patterns, food and water insecurity, and housing instability. The report called for strengthened mitigation and adaptation efforts, improved data and surveillance, locally grounded action, and sustained investment in resilient health systems, emphasizing the need for a more coordinated global public health response (The *Lancet*, UCL Commission 2009) [61].

Drawing on the Commission’s findings, it is noteworthy that while the health impacts of climate change—including those mediated by human mobility—received early attention, migrant health itself remained largely overlooked in climate discourse until much later. By contrast, population movement has long been recognized as a major consequence of climatic change. As early as 1990, the IPCC warned that climate shifts could drive displacement, urbanization, and cross-border migration, whether through sudden-onset disasters or slow-onset crises such as water scarcity and food insecurity (IOM 2008) [62]. Yet, while climate-induced migration was increasingly recognized, its scale and implications remained uncertain due to the complex interplay of financial, social, and structural factors.

Taken together, these developments revealed a structural asymmetry in the evolution of global discourse. Climate change was increasingly framed as a systemic public health threat, and human mobility was acknowledged as one of its foreseeable consequences. Yet institutional responses largely evolved within sectoral silos. Health policy frameworks concentrated on disease burdens and social determinants; migration governance focused on population movement and protection; and climate policy prioritized mitigation and adaptation—without systematically integrating mobility as a determinant of health system resilience. This divergence reflected the absence of a unifying analytical lens capable of conceptualizing climate change, human mobility, and health systems as interacting components within a shared and dynamic risk architecture.

McMichael et al. (2012) [63] were among the first to explore the climate–migration–health nexus, highlighting that migration can function as an adaptive strategy when supported by sound policies and investments. Their analysis implicitly positioned mobility not merely as a demographic outcome of environmental stress, but as a mediating variable within a broader risk and resilience framework. They cautioned, however, that climate-driven migration—particularly to impoverished urban areas—often heightens health risks, especially in low- and middle-income countries. Consistent with the UNDP *Human Development Report* (2009) [64], they emphasized the need for better living conditions, equitable healthcare access, and adaptive public health strategies to protect migrant health and support development. This perspective situates migration within the social determinants of health paradigm, underscoring how structural inequalities condition whether mobility mitigates or amplifies climate-related health risks. However, this recognition was not consistently reflected in climate adaptation instruments. Earlier adaptation planning mechanisms, established under the UNFCCC in 2001 to address urgent climate impacts in Least Developed Countries (LDCs), including the *National Adaptation Programmes of Action* (NAPAs) and the subsequent *National Adaptation Plan* (NAP) process launched in 2010, have largely focused on immediate sectoral priorities—such as water scarcity, agriculture, and disaster risk reduction—while migration dynamics and particularly migrant health have remained comparatively under-addressed.

Addressing this gap required intersectoral collaboration, coordinated policy responses, and stronger recognition—both politically and financially—of migrant health needs. As limitations of sector-specific approaches became increasingly evident, discourse evolved from treating migration as an episodic pressure on health systems to recognizing mobility as a structural determinant within population health governance. In this regard, McMichael and colleagues also referenced *the First Global Consultation on Migration Health* (WHO–IOM, Madrid, 2010) [65], which marked an early effort to address these challenges by calling for systematic monitoring of migrant health, strengthened policy frameworks, and the development of migration-responsive health systems through cross-sectoral partnerships. The concept of *migrant-sensitive health systems*, which emerged from the Madrid consultation and was initially focused on culturally competent, facility-based care, has since evolved into a broader migration-responsive approach that integrates human mobility into public health policies, interventions, and research. This evolution reflects a gradual theoretical reorientation from service adaptation toward systems-level responsiveness. It laid the foundation for linking *migration-sensitive and climate-resilient health systems*, a concept further developed in the following decade and explored later in this paper.

In the years that followed, the climate–health and migration–health agendas largely evolved in parallel, with limited integration. This reflected a pattern in global governance whereby institutions, funding mechanisms, and expert communities developed separately, each defining the problem and designing interventions within their own sectoral boundaries. The global financial crisis of 2007–2008 and its prolonged socio-political effects contributed to reduced policy attention and investment in migrant inclusion in several high-income countries, slowing progress in the migration–health agenda. Within the climate domain, the Fifth Assessment Report of the *Intergovernmental Panel on Climate Change* (IPCC AR5, 2014) [66]—then the most comprehensive global assessment—examined the impacts of climate change on health and on migration largely as separate issues, without explicitly articulating their intersection as a coherent climate–migration–health nexus. The absence of integrated framing reinforced the treatment of mobility as an outcome variable rather than as a determinant shaping exposure, vulnerability, and adaptive capacity.

Nevertheless, building on the growing recognition of health within climate discourse, WHO convened its *First Global Conference on Health and Climate Change* later in 2014, held alongside UNFCCC COP processes. The conference emphasized health system resilience, the protection of vulnerable populations, and the right to health within emerging global climate commitments. The resilience framing marked an important conceptual shift, positioning health systems not only as service providers but as adaptive institutions within complex socio-ecological systems. In 2015, WHO introduced the *Operational Framework for Building Climate-Resilient Health Systems* [12], supporting countries—including least-developed countries—integrating health into *National Adaptation Plans* (NAPs). The framework outlines ten core components, including governance, workforce development, risk monitoring, research, sustainable technologies, and emergency preparedness, aimed at strengthening health systems’ capacity to manage climate risks. In 2016, the WHO *Second Global Conference on Health and Climate Change* [67] further refined these priorities, with increased attention to climate-risk integration, governance, infrastructure resilience, and environmental health.

Despite these advances, migration and mobility were not systematically integrated into climate–health strategies and remained peripheral to their core priorities. A marginalization that illustrates a persistent conceptual divide between environmental risk governance and mobility governance, even as empirical realities increasingly linked them. This began to evolve with the adoption of the 2016 *New York Declaration for Refugees and Migrants*, which—within a broader rights-based, socio-economic development in line with the *Sustainable Development Goals* (SDGs), and humanitarian frameworks—served as an important enabling step. While not explicitly focused on climate change, it helped create normative and political space for more systematic consideration of human mobility and health in contexts increasingly shaped by environmental and climate-related stressors. In this sense, the Declaration functioned as a normative bridge, expanding the governance architecture within which a climate–migration–health nexus could later be articulated.

### 3.1. Post-2016 Global Policy Developments: Advancing Migration and Health on the International Agenda

Amid growing migration crises worldwide, world leaders convened at the 71st UN General Assembly in 2016 to address large-scale refugee and migrant movements. On 19 September 2016, they adopted the *New York Declaration for Refugees and Migrants* [68], reaffirming commitments to strengthened international cooperation, responsibility-sharing, and the protection of refugees and migrants in contexts of displacement and mobility. This political declaration marked a shift from ad hoc crisis response toward a more structured global governance architecture for human mobility.

It laid the groundwork for the 2018 adoption of the *Global Compact for Safe, Orderly and Regular Migration* (GCM) and the *Global Compact on Refugees* (GCR), which recognized climate change, disasters, and environmental degradation as structural drivers of migration. In governance terms, these Compacts institutionalized mobility within a development and risk-management paradigm, reframing migration as a phenomenon requiring anticipatory, cooperative, and whole-of-government, whole-of-society responses. However, while they acknowledged environmental drivers, they did not explicitly conceptualize the interdependencies between climate risk, mobility patterns, and health outcomes within a unified analytical framework. Yet, during this period, the migrant health agenda gained momentum with key milestones such as the *Second Global Consultation on Migration Health: Resetting the Agenda* (IOM-WHO 2017) [69], the World Health Assembly resolutions promoting the health of refugees and migrants in 2017, the UCL-*Lancet* Commission on *Migration and Health* report [70], and the formulation of the WHO Global Action Plan to Promote the Health of Refugees and Migrants (2019–2030). Collectively, these initiatives advanced a rights-based, equity-oriented approach to migrant health and strengthened the normative foundation for migration-responsive health systems.

This policy maturation was paralleled by growing scholarly concern that, despite normative and institutional advances, the analytical foundations of the climate–migration–health relationship remained underdeveloped. Hunter, McMichael et al. (2018) [71] once again observed that, although significant progress had been made in understanding the health impacts of climate change and the links between climate variability and migration, the combined climate–migration–health nexus remained insufficiently explored. They highlighted the need for more integrated evidence capable of informing policies that simultaneously address mobility dynamics, climate vulnerability, and health system capacity. Implicit in their critique was the recognition that climate exposure, mobility decisions, and health outcomes operate as interdependent processes rather than as sequential or isolated events.

Schütte, Gemenne et al. (2018) [72] similarly cautioned that, in the absence of an integrated analytical framework, research trajectories risk fragmentation and policy responses may remain incoherent. They warned that disciplinary silos—between environmental sciences, migration studies, and public health—could reproduce governance silos, thereby limiting the effectiveness of policy interventions. Their call for alignment of data systems, methodologies, and conceptual approaches reflected an emerging systems perspective, recognizing that complex socio-environmental challenges require coordinated analytical architectures.

Building on these calls, Schwerdtle et al. (2018) [73] provided a more comprehensive examination of the climate–migration–health nexus through case studies capturing diverse climate stressors, migration pathways—including immobility, forced displacement, and planned migration—and associated health impacts. By incorporating immobility alongside displacement and migration, they implicitly broadened the analytical lens to include differential adaptive capacities and structural constraints. They underscored the importance of strengthening health systems through universal health coverage, climate resilience, and migrant inclusivity. In line with the outcomes of the *Second Global Consultation on Migration Health*, co-organized by IOM and WHO in 2017 in Colombo, international guidance emphasized integrating migration and health into broader system reforms, promoting multisectoral collaboration, and aligning these efforts with wider development and migration governance agendas.

Similar perspectives were advanced by Villa and Raviglione (2019) [74], who highlighted the need for adaptive and mobility-aware health systems capable of ensuring equitable access to care while systematically integrating migration considerations into health policy and planning.

Collectively, this body of work signaled a gradual epistemic shift—from viewing climate change, migration, and health as intersecting topics to conceptualizing them as components of a dynamically interacting system requiring integrated governance and evidence generation.

Schwerdtle et al. (2018) [73] further demonstrated that environmental and climatic stressors operate primarily as indirect drivers of mobility, amplifying pre-existing social and economic vulnerabilities rather than acting as isolated triggers. This aligns with a political-determinants perspective, in which exposure, adaptive capacity, and health risk are shaped by structural inequalities. They identified a spectrum of associated health outcomes—including infectious and non-communicable diseases, food insecurity, heat-related illness, mental health conditions, and barriers to healthcare access—and underscored the need for rights-based governance approaches linking health, migration, and development. While acknowledging migration as a potential adaptive strategy, they emphasized that its health consequences—protective or adverse—remain highly context-dependent and insufficiently examined, reinforcing the need for policy-relevant, integrated evidence.

Building on this reasoning, McMichael (2020) [10] highlighted the dual vulnerability of those who migrate and those who are unable to move due to economic or social constraints. This further expanded the analytical lens to include immobility as a critical dimension of climate-related health risk. She noted that climate-related mobility will disproportionately affect low-income regions already burdened by fragile health systems and high disease prevalence. Additionally, empirical evidence, such as heat-related deaths among Nepali migrant construction workers in Qatar (Pradhan et al., 2019) [33], highlighted how labour migration in climate-exposed industries can produce preventable harms when extreme heat, employment conditions, and inadequate regulatory safeguards converge. In such contexts, mobility is inseparable from occupational health governance and labour protection systems.

In 2020, Schwerdtle, McMichael and colleagues [75] published a landmark systematic review of literature from 1990 to 2018. Analyzing 1904 studies, with 50 included in the final synthesis, they concluded that although climate–health and climate–migration linkages had been widely examined, their combined interactions remained under-theorized and weakly operationalized. The authors warned that climate change threatens progress in health equity and human rights unless migrant health is more explicitly embedded within climate responses. Central to their conclusions was a call for systems-based approaches connecting climate adaptation, migration governance, and health policy within a coherent public health framework. Migration was framed simultaneously as a determinant of health and, in some contexts, as a form of adaptation—while recognizing that mobility is not universally accessible. The authors advocated for *climate-resilient, migrant-inclusive health systems* aligned with *Universal Health Coverage* and the SDG commitment to leaving no one behind. They also identified persistent data and methodological gaps, highlighting the need for integrated surveillance systems and transdisciplinary research capable of informing anticipatory governance.

In a complementary 2020 synthesis of policy recommendations, Schwerdtle and colleagues [76], distilled six recurring themes across the literature. These included: (i) avoiding the instrumentalization of migration as a universal adaptation strategy; (ii) preserving social and cultural continuity to support resilience; (iii) ensuring meaningful migrant participation; (iv) strengthening health systems and removing structural barriers to care; (v) addressing social determinants of health through cross-sector collaboration; and (vi) integrating health into climate-related loss and damage assessments, with attention to both mobile and immobile populations. Collectively, these themes articulate core governance principles: equity, participation, systems strengthening, and policy coherence across sectors.

The authors further emphasized differentiated vulnerability—highlighting women, older persons, and persons with disabilities—and argued for embedding health within climate loss and damage discussions under the *Warsaw International Mechanism for Loss and Damage* established by COP 19 in 2013. By recognizing non-economic losses, including physical and mental health impacts, this framing broadened climate accountability beyond infrastructure and economic metrics. Subsequent policy developments, including WHO’s 2022 Policy Brief [77] on *Health and Loss and Damage*, reinforced this trajectory.

At the 26th *United Nations Climate Change Conference* in 2021 (COP26), health considerations—including migrant health (WHO, IOM, *Lancet* Migration 2021) [78]—gained unprecedented visibility within climate negotiations. This occurred in the shadow of the COVID-19 pandemic, which exposed structural inequities affecting mobile and marginalized populations and demonstrated the systemic consequences of neglecting inclusive health governance. In this context, the *Alliance for Transformative Action on Climate and Health* (ATACH) [79], was launched to support country commitments by fostering coordination, knowledge exchange, and accountability, and embedding health more systematically within climate adaptation and mitigation processes.

When adapted to explicitly account for human mobility, the ATACH policy and operational tools (Box 5) provide practical entry points for integrating migration considerations into climate-resilient health systems. Such adaptation entails extending resilience planning beyond territorially defined populations to include mobile, displaced, and transit groups. This requires aligning service delivery, preparedness planning, workforce capacity, and surveillance systems with dynamic population movements. In this way, ATACH can function as an institutional platform for operationalising a more explicit climate–migration–health nexus approach.

Box 5 ***Alliance for Transformative Action on Climate and Health*: core focus.**

 A core focus of ATACH is supporting countries to advance climate-resilient health systems through:

- conducting *Climate and Health Vulnerability Assessments* (V&As): Evaluating risks at both population and healthcare facility levels.
- developing *National Health Adaptation Plans* (HNAPs) aligned with broader *National Adaptation Plans* (NAPs) and climate policies, on the basis of *V&A* findings.
- Improving access to climate finance through mechanisms such as the *Global Environmental Facility, Green Climate Fund, and Adaptation Fund*, and other funding mechanisms.

This trajectory was reinforced at COP26, where the International Organization for Migration, World Health Organization, and The *Lancet*- Migration issued joint statements [80], calling for integrated responses to the intersecting challenges of climate change, health, and human mobility. Priority actions highlighted included: incorporating human mobility into *National Adaptation Plans* and *Nationally Determined Contributions*, strengthening migrant-inclusive health systems, promoting community-led climate adaptation, and mobilizing predictable, sustainable financing. Complementary funding initiatives—such as the UNHCR *Climate Resilience Fund* (UNHCR 2021) [81] and the *Global Cities Fund for Migrants and Refugees* (2021) [82]—illustrated emerging efforts to translate nexus principles into resource allocation and local implementation mechanisms. These developments indicate a gradual transition from simply recognizing the Climate-Migration-Health link in principle, to actively testing and implementing institutional approaches. At the same time, the *Lancet* Migration European Regional Hub (2021) [83] highlighted that significant gaps remained in both evidence and financing, particularly in regions disproportionately affected by climate-related and planetary health emergencies. The emphasis was therefore not simply on generating more research, but on producing policy-relevant knowledge—research that can directly inform coordinated interventions across sectors and governance levels, bridging the gap between conceptual acknowledgment and actionable, multisectoral responses.

Another topic highlighted by the review concerns the use of quantitative evidence and digital health systems. Traditional qualitative approaches often struggle to capture the complex, evolving links between climate, migration, and health. As Schwerdtle et al. note (2020) [75], analyses should be complemented by data-driven, systems-oriented methods. For example, Reuveny (2021) [84] developed a *Dynamic Simulation Model* (DSM) that combines climate impacts, health indicators, migration flows, and conflict risks, allowing scenario testing, forecasting, and policy evaluation—tools that help governments anticipate and plan for emerging risks.

The effectiveness of these tools depends on linking them to decision-making processes, multisectoral coordination, and community engagement. Improving interoperability between climate data, migration statistics, and health surveillance systems can strengthen risk assessment and adaptive capacity, consistent with Objective 1 of the *Global Compact for Safe, Orderly and Regular Migration*, which calls for accurate, comparable data to guide evidence-based migration governance. Integrated approaches that combine public health analysis, systems thinking, and governance perspectives are therefore essential for turning data into actionable policies and resilient responses.

### 3.2. Recent Intensification of Research on the Climate–Migration–Health Nexus

Building on Schwerdtle et al.’s 2020 review [75], which identified only 50 empirical studies published between 1990 and 2018, McMichael’s 2023 narrative review [85] documents a notable expansion of the evidence base. Analyzing 36 empirical studies published between 2018 and 2022, the review reflects growing scholarly attention to the interconnections between climate change, human mobility, and health.

Beyond documenting expansion, the literature reveals several conceptual shifts. Mobility is increasingly conceptualized as embedded within broader social and ecological systems rather than as a direct or isolated consequence of climatic events. Climate influences mobility and health not in isolation, but through its interaction with economic precarity, governance conditions, social inequalities, and conflict. In synthesizing recent evidence, McMichael [85] underscores the multidimensional nature of these linkages and the need for integrated health responses to climate-related mobility.

At the same time, attributing migration directly to climate remains methodologically challenging, as mobility is rarely driven by a single factor. While some studies examine whether climate-related stressors—such as reduced food and water availability—directly influence migration decisions, findings remain mixed (Schütte et al. 2018) [72]. Nonetheless, empirical evidence consistently identifies elevated health risks among climate-affected migrants, including limited access to healthcare, increased exposure to infectious diseases, mental health stressors, threats to sexual and reproductive health, and food insecurity (McMichael 2023) [85]. These risks arise during transit as well as after settlement, indicating that vulnerability extends beyond the point of displacement.

Although much of the literature available concentrates on displacement, the more analytically relevant issue concerns how climate-related stressors interact with social, economic, and political systems to shape patterns of vulnerability and resilience along diverse migration pathways (Schütte et al. 2018) [72]. Migrant workers, for example, often experience disproportionate exposure to extreme heat and other hazards, including documented severe heat stress (Pradhan 2019) [33] and insufficient occupational health preparedness in climate-sensitive sectors such as agriculture and construction (Messeri et al., 2019) [86]. These findings reinforce a structural understanding of risk, in which living conditions, regulatory gaps, and deficits in social protection mediate health outcomes. Accordingly, climate is increasingly framed as a risk multiplier, reinforcing the policy imperative to protect health and rights irrespective of specific migration triggers.

Collectively, these findings point to migrants’ heightened exposure to climate-related risks and, in many contexts, their constrained adaptive capacity (Romanello et al. *Lancet* Countdown Report, 2021) [87]. Rather than treating these vulnerabilities as inherent to mobility, the literature situates them within structural conditions that shape access to resources, services, and protection. In this regard, McMichael [85] highlights the role of health systems in mitigating risk through targeted interventions for vulnerable populations, strengthened disaster preparedness and response, and cross-sectoral action on the social determinants of health.

Framing climate as a *risk multiplier* also reshapes how adaptation is understood. If vulnerability emerges from the interaction between climate stressors and structural inequalities (Schütte et al. 2018) [72], adaptation cannot be reduced to short-term coping measures. Rather, it requires structural and multisectoral responses. Intersectoral collaboration and comprehensive social protection are identified as central to building resilience and enabling more durable change in the context of climate-related mobility (Tenzing, 2020) [88]. In this perspective, adaptation involves addressing underlying determinants of health vulnerability—including labor conditions, legal status, housing, social and gender norms, and access to essential services—while strengthening health systems’ capacity to respond (McMichael, 2023) [85].

Community participation is frequently highlighted as a component of adaptive capacity. However, the literature cautions against overidealizing local action. While migrant and mobile populations may possess valuable adaptive knowledge and practices, these capacities often remain constrained by limited legal recognition, institutional inclusion, social acceptance, and financial resources. As Barnett underscores (2022) [89], community-led initiatives can strengthen resilience but are insufficient in the absence of supportive structural policies. Without systemic backing, adaptation risks shifting responsibility for climate response onto already marginalized groups. Climate justice–oriented approaches therefore call for adaptation frameworks that explicitly address underlying inequalities through social protection, secure livelihoods, legal safeguards, and inclusive governance. Such approaches link community-level initiatives to national and international policy mechanisms capable of enabling sustainable and climate-resilient mobility pathways.

These findings are consistent with advocacy advanced by the *United Nations Network on Migration* (UNNM) at COP27 in 2022 [90], which emphasized rights-based climate policies recognizing migrants as rights holders and promoting social justice. The Network calls for the integration of safe and regular migration pathways into climate adaptation strategies, including mechanisms that support labor mobility, uphold human rights, facilitate education and family reunification, and introduce flexible visa arrangements to enhance resilience to climate change. It further encourages bilateral and regional agreements, as well as tailored visa categories, to respond to the needs of climate-affected populations, with particular attention to accessibility for women and children. Recent scholarship reinforces this direction. Silenzi et al. (2023) [11] argue that both climate adaptation policies and migration governance frameworks should systematically incorporate health objectives, with explicit attention to reducing health inequities.

From a public health perspective, this evolving body of literature—largely composed of conceptual analyses, interpretive syntheses, and normative arguments rather than consolidated longitudinal evidence—points toward the need for more integrated policy architectures. Specifically, it underscores the importance of aligning climate adaptation, migration governance, and health system strengthening within coherent and operational frameworks. Such alignment would facilitate the translation of emerging knowledge into coordinated policy development, multisectoral decision-making, and implementation processes, while centering equity, protection, and health outcomes for climate-affected populations.

Financing remains a critical constraint in advancing migrant health within climate adaptation strategies. McMichael (2023) [85] underscores the urgent need for more equitable funding to integrate migrant health into climate adaptation efforts, both to promote social justice and to strengthen resilience among climate-affected populations. Despite growing recognition of health adaptation needs and initiatives addressing loss and damage, funding—particularly for interventions related to human mobility—remains insufficient.

In recent years, health has gained greater visibility within the climate finance agenda. High-level commitments—such as the endorsement of guiding principles for climate and health financing at COP28 (2023) [91], —and the growing inclusion of health in *Nationally Determined Contributions* (NDCs) and adaptation planning signal progress in agenda-setting and multisectoral engagement. However, this momentum has not translated into proportional financial allocations. While more than 90% of countries now include health priorities in climate plans and strategies, global adaptation finance remains critically insufficient relative to identified needs, and even where health is referenced, dedicated funding is limited. The 2025 *Lancet Countdown on Health and Climate Change* (Romanello et al. 2025) [92] highlights that climate adaptation finance continues to fall far short of projected health adaptation needs, and that investment in adaptation—crucial for protecting health—is uneven and underfunded relative to both the scale of risk and national cost estimates. Specifically, the report shows that key climate finance flows, such as those tracked from the *Green Climate Fund* and multilateral banks, remain small compared with the billions of dollars in health adaptation costs that countries have disclosed in their adaptation planning processes.

Consistent with this, previous analyses have noted that only about 0.5% of multilateral climate finance is directed to projects that explicitly address human health and that a very small share of adaptation funding targets health systems and resilience explicitly (*Green Climate Fund* 2024 [93]; WHO, COP30, 2025 [94]). This underscores the persistent gap between rhetorical recognition of health in climate frameworks and the reality of resource allocations and emphasizes the need for intentional policy design and financing mechanisms that specifically support health-related adaptation and resilience building in climate–migration–health contexts.

Policy processes under the *Warsaw International Mechanism for Loss and Damage*, including its *Task Force on Displacement*, have repeatedly called for increased resource mobilisation to minimize loss and damage associated with climate-related mobility (Platform on Disaster Displacement, 2024) [95]. Yet, the extent to which climate finance specifically addresses the health needs of mobile populations remains poorly documented, highlighting the need for improved tracking and accountability mechanisms.

Overall, the literature highlights a persistent misalignment between policy recognition and financial allocation for mobile populations. Additionally, while displacement is increasingly acknowledged within climate finance discussions, other forms of climate-related mobility—such as labour migration and planned or adaptive migration—remain comparatively under-addressed. As McMichael (2023) [85] argues, more systematic integration of health into adaptation and loss and damage financing—including investments in climate-resilient and migration-sensitive health systems, as well as cross-sectoral strategies to mitigate health risks—is essential to ensure equitable protection and resilience for both mobile populations and those left behind.

Across recent literature, three interlinked themes consistently emerge as central to addressing climate-related health risks among mobile populations: (i) generating robust evidence, (ii) developing context-specific interventions, and (iii) translating research into policy and practice.

On the first theme, several studies highlight persistent gaps in understanding the complex interplay between climate, migration, and health. Issa et al. (2023) [96] identify research gaps regarding internal displacement, immobile populations, and vulnerable groups in high-risk regions, while emphasizing the need for interdisciplinary collaboration, strengthened data collection, and enhanced education for policymakers and clinicians. Funding limitations, particularly in low- and middle-income countries, further constrain knowledge generation, reinforcing the role of global networks and partnerships, such as the *CliMigHealth* International Thematic Network (*CliMigHealth* is an international, transdisciplinary network, integrating diverse expertise and better representing the Global South, through strengthening research, educational and knowledge translation capacity) and partnerships fostered by WHO, IOM, and *The Lancet* at COP26 (2021) [80].

The second theme—context-specific interventions—is addressed through evidence showing how health outcomes among climate-affected migrants are shaped by intersecting social, demographic, geographic, and political determinants (Khalid et al. 2023 [97]; (Bharadwaj and Huq 2022) [98]. Schwerdtle et al. (2024) [99] highlight that while climate impacts on health are widely acknowledged, operational adaptation solutions—particularly in fragile and conflict-affected settings—remain underdeveloped. Their *Adaptation Continuum* framework situates responses along a spectrum from maladaptation to resilience-building and aligns with the *Humanitarian–Development–Peace Nexus*, emphasizing proactive, resilience-oriented strategies, in line with policy guidance for integrated climate adaptation, humanitarian action, development, and peacebuilding (Pörtner, H.O. et al.; IPCC 2022) [100].

The third theme—translating evidence into policy and practice—emerges from normative and policy-oriented literature. The right to the highest attainable standard of health for migrants is emphasized (Severoni et al. 2024) [101] alongside calls for climate-resilient health systems that address the specific vulnerabilities of displaced populations. Policy analyses informing COP29 and the WHO *Global Research Agenda on Health, Migration and Displacement* (WHO 2023) [102] reflect a gradual shift from short-term emergency responses toward integrated, proactive strategies grounded in evidence, strategic planning, and long-term investment. Despite encouraging progress, WHO’s recent review of 95 health-system interventions (WHO 2025) [103] shows persistent structural gaps in governance, financing, and health information systems, exposing the implementation gap between recognition of the nexus and effective action.

Overall, the review highlights the importance of cross-sectoral and interdisciplinary collaboration, community engagement, and the adaptation of existing interventions to evolving population needs. It also identifies substantial evidence gaps, particularly regarding long-term and sustainable interventions, mitigation strategies, system-wide approaches, and impact evaluation, as well as limited data from highly climate-vulnerable regions such as the MENA. Addressing these gaps is critical for advancing migrant-inclusive, climate-resilient health systems and requires systematically applying a migration lens to climate-resilient health agendas and a climate lens to migration health efforts, moving beyond predominantly reactive responses. The mapping of concepts and emerging evidence underscores that the climate–migration–health nexus is not defined by linear causality but by structurally interacting processes. Climate change functions as a risk multiplier, interacting with pre-existing socio-economic and political fragilities. Human mobility serves both as an adaptive strategy and a source of differential vulnerability, depending on governance conditions and access to resources. Health systems operate as institutional mediators that can either buffer or amplify these intersecting pressures.

Rather than discrete policy domains, climate adaptation, migration governance, and health system resilience emerge as interdependent components of a shared systems dynamic. This structural interdependence provides the theoretical foundation for the Nexus Action Framework, which seeks to organize these interacting determinants within a coherent operational architecture.

## 4. From Evidence to Action: Framing a Nexus Action Framework for Climate, Migration, and Health

The reviewed literature demonstrates broad convergence on the interconnected nature of climate change, migration, and health, the multiple entry points through which this nexus can be addressed, and the need for more structured and coordinated responses. Across both peer-reviewed and grey literature, studies frequently highlight fragmented approaches and limited operational coherence in translating the nexus into policy and practice. These challenges are particularly evident in regions such as the Middle East and North Africa (MENA), where climate-related mobility and health vulnerabilities are intensifying.

Despite the growing visibility of the climate–migration–health nexus in global and regional policy discourse, much of the literature tends to focus on determinants, impacts, and policy linkages, with comparatively less attention to sustained and coordinated implementation. Recent work led by the World Health Organization represents an important step toward greater operationalisation, particularly from a health-systems-strengthening perspective through the promotion of *migrant-inclusive and climate-resilient health systems* (WHO, 2025) [104]. Beyond the health sector, however, implementation approaches remain uneven, and integration of the nexus across policy domains varies considerably. These dynamics suggest the potential value of a more consolidated, multisectoral action framework that could offer common orientation across climate adaptation, migration governance, development, humanitarian response, and health systems, while remaining adaptable to diverse contexts and levels of evidence.

**Figure 1:**
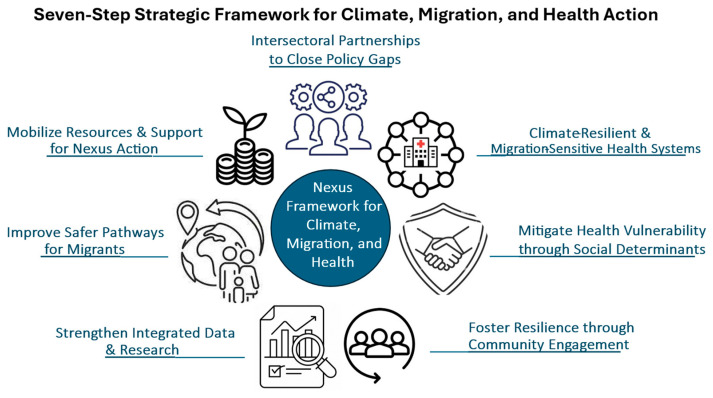
Core Components for Operationalising the Climate–Migration–Health Nexus.

Within this framing, climate-resilient and migration-sensitive health systems are conceptualised not as an endpoint but as a central organising axis for aligning multisectoral action. Progress depends on coherent linkages across domains such as climate action, migration governance, social cohesion, community engagement, data and technology systems, and related development and health priorities, rather than isolated or sector-specific interventions. Building on this perspective, the following section proposes seven interrelated components visualized in Figure 1, intended to support the operationalisation of the climate–migration–health nexus as a more coherent and actionable policy domain.

These components are intended as a structured yet adaptable reference to guide alignment across research, policy, and practice and are described in detail in the following sections. The framework has informed ongoing action-oriented technical work led by the International Organization for Migration (IOM) Regional Office for MENA (2025) [105], in partnership with governments, partner agencies, and other stakeholders. The contribution of the proposed Nexus Action Framework lies not in introducing new sectoral concepts, but in operationalising their integration—offering a structured, policy-relevant orientation to align climate action, migration governance, and health system strengthening within a single, adaptable framework.

The pillars are designed to reflect both the directional pathways linking climate exposure to mobility patterns and health outcomes, and the feedback dynamics through which governance arrangements, financing structures, and service responses can either mitigate or exacerbate these effects over time.

1. *Bridging Policy Gaps through Collaborative Partnerships for Global Action*

Addressing the climate–migration–health (CMH) nexus requires overcoming fragmented policies and siloed approaches through strategic, cross-sector partnerships. Effective action depends on recognizing and addressing structural determinants—including political economy constraints, regulatory environments, labour market structures, and migration governance regimes—which shape both implementation capacity and equity outcomes.

Mechanisms exist within climate action, migration governance, and health to assess progress and foster commitments, including health-specific frameworks such as the WHO *Global Action Plan to Promote the Health of Refugees and Migrants* presented by WHO to the World Health Assembly in 2019, covering the period 2019–2030, the *International Migration Review Forum* (IMRF), and advocacy initiatives within UNFCCC processes. While inter-agency statements and advocacy initiatives have raised the profile of the CMH nexus, efforts to translate these commitments into coordinated action remain limited and largely sector-specific. Strengthened and more systematic approaches are needed, including the establishment of structured pathways or dedicated tracks for CMH within global and regional processes.

Bridging these gaps requires leadership and stewardship by governments, supported by intergovernmental agencies, civil society, and other key stakeholders. In this context, governments endorsing the *Rabat Declaration* at the *Third Global Consultation on the Health of Refugees and Migrants* (WHO–IOM–UNHCR and Government of Morocco 2023) [106], which commits to improving the health of mobile people and host communities, are well positioned to champion whole-of-government and whole-of-society approaches. Engaging actors across local, national, regional, and global levels can support the alignment of priorities, strengthen data sharing, and enable the development of adaptable, forward-looking policies. Cross-sector partnerships are essential to foster shared understanding, mobilize flexible and sustainable financing, and embed CMH considerations within key global policy processes, including the UNFCCC, the Global Compacts on Refugees and Migrants, the *Sustainable Development Goals*, relevant health frameworks, and post-2030 successor agendas.

Institutionalizing integrated governance approaches across these levels is critical to delivering coordinated, actionable, and durable solutions that enhance resilience, equity, and system-level preparedness, both in the MENA region and beyond. By linking partnerships with policy integration, this pillar illustrates how collective stewardship and multisectoral collaboration can bridge existing gaps and strengthen the operationalization of the climate–migration–health nexus. Examples of emerging efforts to address policy gaps at the intersection of climate change, migration, and health in the MENA region are summarized in Box 6.

Box 6 **Bridging Policy Gaps in MENA.**

In the MENA region, although structured mechanisms explicitly integrating climate, migration, and health remain limited, recent interregional consultations on refugee and migrant health (WHO, 2023) [103] and joint climate–health programming initiatives in countries such as Jordan (IOM–WHO–UNDRR, 2024) [107] illustrate incremental efforts to foster cross-sector dialogue and coordination relevant to the CMH nexus.

2. *Strengthening Climate-Resilient and Migration-Sensitive & Responsive Health Systems:*

The relationship between climate stressors, mobility patterns, and health system performance is dynamic rather than linear; system strain may exacerbate vulnerabilities, contribute to onward or secondary mobility, and further intensify service demands in receiving areas. Adapting health systems to the intersections of climate change, human mobility, and health is increasingly recognized as a key area of research, policy, and operational interest, and represents a central pillar of the proposed *Climate–Migration–Health Nexus Action Framework* (NAF-CMH). Climate impacts affect both populations on the move and those unable or unwilling to migrate, underscoring the importance of the mobility–immobility continuum for public health planning and response.

The WHO concept of *climate-resilient health systems* (WHO, 2015) [12] provides a foundational architecture, comprising six building blocks—leadership and governance, health workforce, health financing, health information systems, service delivery, and access to essential medicines and technologies—which have been adapted to incorporate climate considerations. Building on this foundation, the NAF-CMH draws on insights from peer-reviewed literature, grey and institutional sources, and operational experience to visualize how climate, mobility, and health intersect across health system components, while explicitly aligning with global development and climate platforms. Table 2 presents the climate-resilient and migration-sensitive adaptation of each building block, while Table 3 provides concise explanatory descriptions.

This presentation does not prescribe specific actions but outlines key functional domains for climate-resilient and migration-sensitive health systems, highlighting areas of policy attention, system capacity, and evidence generation relevant to climate- and mobility-affected contexts.

It is important to recognize that literature explicitly addressing the nexus of climate, mobility, and health systems is still limited, particularly in region-specific contexts. The framework reflects the authors’ interpretation of existing evidence, synthesizing available studies, policy documentation, and operational practice to provide a conceptual overview. It is intended as a tool for discussion, knowledge exchange, and operational reflection, rather than a normative or prescriptive guidance. By linking climate, mobility, and health considerations to established health system components, the framework provides a structured visualization of emerging intersections, highlighting potential areas for attention, research, and coordination in diverse contexts. It emphasizes flexibility and adaptability, recognizing that health system responses must be shaped by local realities, available evidence, and evolving climate and mobility challenges.

Although this paper does not elaborate in detail on structural and service-delivery dimensions, it underscores that psychosocial and mental health support are essential components and a foundational element of climate-resilient and migration-sensitive health systems. Populations exposed to climate-related shocks, protracted environmental stress, displacement, and conflict face elevated risks of psychological distress, trauma-related disorders, anxiety, and depression, particularly in conflict-affected and displacement settings (Figueiredo et al. 2024) [108]. These risks are often compounded by social exclusion, legal precarity, disrupted livelihoods, and barriers to accessing care. A climate-resilient and mobility-responsive health system must therefore integrate mental health and psychosocial support (MHPSS) across the continuum of care—through primary health care platforms, community-based services, referral systems, and coordination with social protection and protection actors. Evidence from policy and regional analyses highlights the importance of structured screening, culturally responsive interventions, and institutional support mechanisms for displaced and environmentally vulnerable populations. Embedding MHPSS within preparedness, response, and long-term adaptation strategies strengthens system resilience, promotes equity, and enhances the capacity of health systems to address climate-induced mobility, displacement, and involuntary immobility.

Examples of emerging efforts toward climate-resilient and migration-sensitive health systems in the MENA region are summarized in Box 7.

Box 7 **Resilient and Migration-Sensitive Health Systems in MENA.**

In the MENA region, several countries have begun integrating climate resilience into health sector planning through national adaptation strategies and health components of *National Adaptation Plans* (e.g., Jordan Ministry of Health, 2025) [109]; WHO-RMRO 2025 [110] while refugee-hosting contexts such as Jordan and Lebanon (MOPH Lebanon, 2023) [111] continue efforts to strengthen inclusive health service delivery for displaced populations, reflecting gradual movement toward climate-resilient and migration-sensitive health systems.

3. *Mitigating Health Vulnerabilities by Addressing Social Determinants*

The narrative review highlights that climate-related health vulnerabilities linked to migration are shaped primarily by social and environmental determinants beyond the health sector, including access to adequate housing, clean water and sanitation, education, safe and decent work, social protection, and inclusive urban environments across the migration continuum. Migration itself is increasingly recognized as a structural social determinant of health, influencing exposure to climate risks and shaping opportunities to access services and resources.

This NAF component emphasizes the importance of intersectoral action to address these determinants, aligning with global frameworks such as the Sustainable Development Goals (notably SDG 3.d *National and Global Health Risks*, SDG 5 *Gender Equality*, SDG 10 *Reduced Inequalities*, and SDG 11 *Sustainable Cities and Communities*, among other), the *Global Compact for Migration* (particularly Objective 6 *Decent Work*; Objective 7 *Reduce Vulnerability*; Objective 15 *Access to Basic Services*; and Objective 16 *Empower Migrants*), and the *Sendai Framework for Disaster Risk Reduction*. Such alignment situates health equity within broader development, labour, urban, and disaster governance agendas, rather than treating it solely as a health-sector responsibility.

Implementation, monitoring, and accountability mechanisms within these agendas should be adapted to ensure that the climate–migration–health nexus is explicitly embedded in planning, reporting, and evaluation processes. Coordinated, multi-sectoral action—engaging government sectors beyond health, as well as the private sector, academia, and civil society—is essential to reduce climate-exacerbated vulnerabilities and foster inclusive, resilient communities. Mechanisms to track progress, periodically review objectives, and adjust interventions help ensure that CMH considerations are systematically reflected in policies and programs.

Illustrative examples of multisectoral actions addressing social determinants of health for mobile and climate-affected populations in the MENA region are summarized in Box 8.

Box 8 **Addressing Social Determinants for Mobile Populations.**

In the MENA region, multisectoral actions addressing intersecting social determinants of health, mobility, and climate risks can be observed in a variety of contexts. In Jordan, efforts to expand access to safe water, sanitation, and health services in drought-affected and refugee-hosting communities illustrate how basic determinants and service access are being addressed in climate- and mobility-stressed settings (UNICEF & WHO, 2021) [112]. In Lebanon, Jordan, Egypt, and Turkey, joint health and social protection activities under the Regional Refugee and Resilience Plan (3RP)—established in 2015 following the Syria crisis as a regional coordination platform bringing together humanitarian and development partners—seek to improve living conditions and access to essential services for refugees, displaced populations, and vulnerable host communities amid ongoing conflicts, natural and climate-related disasters, and deteriorating socio-economic and humanitarian conditions (UNHCR and UNDP, 2024) [113]. In the *Gulf Cooperation Council* (GCC) context, extreme heat exposure has been documented as a significant health risk for migrant workers, with studies highlighting heightened heat-related illnesses and occupational hazards in countries such as Saudi Arabia, the United Arab Emirates, Qatar, and Kuwait (Human Rights Watch, 2024) [114]. In response, some Gulf states—Bahrain in 2025 among them—have begun adjusting work-safety measures, such as extending midday heat bans and exploring risk-based occupational guidelines, while regional social protection reforms have introduced new insurance and end-of-service benefit schemes for migrant workers across GCC countries (ILO, 2023). [115], reflecting nascent efforts to address climate-exacerbated health vulnerabilities among mobile labour populations.

4. *Strengthening Community Resilience through Participatory Approaches:*

Evidence from research and grey literature underscores the critical role of community-led initiatives in addressing the interconnected challenges of climate change, migration, and health. Locally driven approaches are better positioned to respond to context-specific needs and achieve sustainable impact, enhancing resilience and adaptive capacity among migrants, displaced populations, and host communities. Studies highlight the utility of event-based disease surveillance, mobile health (mHealth) tools, social media engagement, and social cohesion programs in identifying emerging health risks and supporting locally led adaptation.

Direct engagement with affected communities, coupled with continuous feedback and monitoring mechanisms, is essential to ensure interventions remain responsive, equitable, and effective. Embedding participatory processes within broader governance, health, and *humanitarian–development–peace* (HDP) strategies positions communities as active agents of resilience, complementing systemic and policy-level actions while reinforcing social cohesion. In fragile and conflict-affected settings, participatory approaches can also support peacebuilding by reducing climate-related tensions, strengthening trust between communities and institutions, and reinforcing the HDP nexus, where climate change increasingly acts as a threat multiplier for instability and displacement.

Examples of locally led, participatory initiatives that support resilience to climate- and mobility-related health risks in the MENA region are presented in Box 9.

Box 9 **Community-Led Resilience Initiatives.**

Locally led, participatory initiatives in the MENA region have demonstrated potential to enhance resilience to climate- and mobility-related health risks. In Lebanon, community-based early warning systems and outreach programs in informal tented settlements for Syrian refugees have improved disease surveillance and health service uptake (Lyles et al., 2016) [116]. Outreach volunteer networks have also been used to expand primary health care engagement and self-monitoring for chronic conditions among displaced populations, with trained refugee outreach volunteers facilitating health education and referrals tailored to local needs (Sethi et al., 2017) [117] In the same country, community-engaged research involving Syrian refugees has been used to shape mental health and psychosocial support interventions, ensuring that design and delivery reflect community priorities and cultural context (Nakkash et al., 2024) [118]. In Jordan, participatory resource management programmes have brought together local host communities and refugees in dialogue and joint water sector planning to improve access to water and reduce conflict over scarce resources (GIZ, 2025 [119]). Across these examples, participatory approaches facilitate context-specific adaptation, strengthen social cohesion, and complement systemic health and governance measures, aligning with the HDP nexus where climate acts as a multiplier of displacement risks.

5. *Supporting Integrated Data Systems, Innovation, and Research*

The narrative review highlights broad consensus on the importance of evidence-informed decision-making to address the climate–migration–health nexus. Integrated data systems are essential for linking climate impacts (e.g., temperature and rainfall patterns), migration dynamics (e.g., displacement and mobility flows), and health outcomes (e.g., disease incidence, access to care). Disaggregated data—by age, gender, migration status, socio-economic background, and location—enable identification of vulnerabilities and inform targeted interventions. Standardized indicators, innovative data collection methods, and interoperable systems across health, climate, and migration sectors can enhance comparability and policy relevance, while regional platforms could strengthen collaboration, knowledge exchange, and capacity-building across MENA or beyond.

Health surveillance should incorporate migration flows and climate-sensitive risks, such as vector-borne diseases and heat-related illnesses. Tools including GIS, remote sensing, predictive modeling, mobile health (mHealth) applications, one-stop platforms such as the IOM *Migrant Application* (MigApp), and community-based monitoring can support data collection in remote or hard-to-reach populations while fostering participatory engagement.

Further integrated research is needed to examine intersections between environmental change, food and water security, migration, and health, with attention to gender- and context-specific vulnerabilities. Under-researched populations—including irregular migrants, seasonal laborers, nomadic communities, and stateless persons—require targeted investigation to understand exposure, risk, and barriers to services. Additionally, research on policy impacts, financing mechanisms, and the cost of inaction is critical to support long-term resilience, equitable access, and multisectoral decision-making. Strengthening partnerships with academic institutions, research networks, and technical agencies can foster innovation, build expertise, and expand training programs on climate, mobility, and health, supporting evidence-informed planning and coordinated action across local, national, and regional levels. 

Recent initiatives aimed at improving data integration, surveillance systems, and research collaboration relevant to the climate–migration–health nexus are outlined in Box 10.

Box 10 **Integrated Data Systems and Research.**

In the MENA region, initiatives such as the development of the *Migrant Health Country Profile tool* (MHCP-t) to strengthen migrant health surveillance and cross-country data comparability (Evangelidou et al., 2025) [120], the scale-up of electronic health information systems including DHIS2 platforms in Lebanon and communicable and NCD surveillance modules in Jordan (WHO EMRO, 2024) [121], and regional research collaborations on climate and health under the MENA *Hub for Action Research on Climate Change and Health* (IDRC 2025) [122] demonstrate emerging efforts to improve data integration, innovation, and evidence generation relevant to the climate–migration–health nexus.

6. *Enhancing Safer and Healthier Pathways for Labor Migrants and Mobilizing Health Workforce Capacity Among Migrants and Displaced Populations*

Developing effective migration policies is essential for addressing climate-induced mobility. Such policies should establish safe and regular migration pathways, integrate climate considerations into planning, strengthen international cooperation, and address the vulnerabilities of climate-affected populations. Evidence highlights that migration can serve as an adaptive response to climate stress, but it should be treated as a last-resort strategy, complemented by resilience approaches for those unable or unwilling to move.

Safe migration pathways connect immediate humanitarian support with long-term health, development, and adaptation objectives. Policies should ensure migrants have continuous access to healthcare—including pre-departure medical examinations, vaccinations, and services in receiving communities—while linking these pathways to climate- and migration-sensitive health systems (Component 2).

Labor mobility, particularly in sustainable development and the green economy, offers opportunities to support climate-affected populations while meeting broader health and livelihood needs. Integrating displaced or migrant health workers into climate-resilient and green-economy initiatives leverages their expertise, strengthens health system capacity, and promotes individual livelihoods, thereby advancing both environmental and health objectives (Nash, S.; van der Voort, E.C.A 2024) [123].

Examples of initiatives linking safe migration pathways, labour mobility governance, and climate-related health protection are highlighted in Box 11.

Box 11 **Safe Migration Pathways and Workforce Mobilization.**

Safe and regular labour migration pathways can function as adaptive responses to climate stress when grounded in rights-based governance and health protection. In North Africa, *the Towards a Holistic Approach to Labour Migration Governance and Labour Mobility in North Africa* (THAMM) programme in Morocco, Tunisia, and Egypt strengthened regular migration channels, worker protection systems, and skills matching—elements that can support climate-affected populations through safer mobility options (ILO 2019) [124]. In Gulf countries, labour reforms (ILO 2023) [125] improving job mobility and occupational protections—particularly measures addressing extreme heat exposure (Migrant-Rights.Org 2023) [126]—demonstrate how migration governance intersects with climate-sensitive health risks. Aligned with Objectives 2 (Minimize Adverse Drivers), 5 (Regular Pathways) and 15 (Access to Basic Services) of the *Global Compact for Safe, Orderly and Regular Migration*, these approaches illustrate how structured labour mobility schemes, health access across migration pathways, and occupational safeguards can reduce vulnerability, enhance resilience, and contribute to climate-responsive migration governance.

7. *Mobilizing Technical, Financial, and Political Resources to Translate the Nexus Agenda into Action*

Addressing health challenges linked to climate-induced migration requires coordinated mobilization of technical expertise, political commitment, and strategic resources. Evidence underscores that raising awareness, aligning priorities, and building a robust evidence base are critical to guiding actionable interventions at national, regional, and global levels. Early stakeholder mapping, consultations, and concertation processes help capture diverse perspectives, set shared objectives, and ensure sustained community engagement. Cross-sectoral partnerships among government agencies, intergovernmental organizations, civil society, and the private sector are essential for coordinated action. Engaging regional institutions—such as in the case of MENA the *League of Arab States* and the *Gulf Cooperation Council* (GCC)—and consultative platforms like the *Abu Dhabi Dialogue* and the *Arab Regional Consultative Process on Migration and Refugee Affairs*, as well as global forums such as the UNFCCC COP and the Paris Agreement, can enhance the visibility, legitimacy, and uptake of the CMH Nexus agenda.

Integrating migrant health considerations into *National Climate Plans*, conducting vulnerability and resilience assessments, and aligning these efforts with *Nationally Determined Contributions* (NDCs) and *National Adaptation Plans* (NAPs) strengthens national resilience and promotes global health equity. While financing mechanisms for resilient and inclusive health systems are discussed in Component 2, strategic resource mobilization remains essential to ensure the sustainable, long-term implementation of CMH integrated interventions at the global, regional, and local levels.

Existing regional cooperation platforms and climate financing mechanisms may offer important entry points for advancing integrated climate–migration–health priorities; selected examples are highlighted in Box 12.

Box 12 **Mobilizing Resources for Action.**

The World Bank Group’s *Roadmap for Climate Action in the Middle East and North Africa (2021–2025)* (World Bank Group 2021) [127]. aims to mobilize around USD 10 billion in climate-related investments to support resilient development, including climate-smart agriculture, water security, energy transition, and sustainable finance mechanisms.

Complementing such initiatives, multilateral climate finance mechanisms such as the Green Climate Fund (ODI Global 2025) [128]. provide funding for adaptation and mitigation projects across developing regions, including MENA countries, supporting climate-resilient infrastructure, water management, and food-security programmes.

## 5. Conclusions

The MENA region offers a diverse and instructive context in which climate change, human mobility, and health risks intersect within complex social, political, economic, and institutional environments. Climate stressors interact with demographic transitions, urbanization, displacement dynamics, and, in several contexts, protracted conflicts and political instability—shaping both vulnerabilities and response opportunities. In parts of the region affected by armed conflict and humanitarian crises, populations continue to endure profound loss, displacement, and disruption of essential services, with lasting consequences for physical and mental health. Recognizing this human reality is essential to framing policy responses that are not only technically sound but grounded in dignity, equity, and solidarity.

While institutional readiness and resources vary across countries, several have demonstrated leadership in health system development, migration governance, and climate adaptation. These differences highlight the importance of approaches tailored to national and subregional realities. At the same time, the patterns observed in MENA offer insights relevant to other regions facing similarly interconnected climate, mobility, and health challenges.

The *Nexus Action Framework for Climate, Migration, and Health* (NAF-CMH) is proposed as a policy-oriented, integrative tool to navigate this landscape. Rather than introducing a new sectoral model, it structures alignment across climate, migration, and health domains, clarifies entry points for coordinated action, and supports dialogue among institutions that often operate in parallel. By connecting governance, health systems, mobility-sensitive planning, social determinants of health, and evidence generation, the framework offers a shared reference for prioritizing actions and reducing fragmentation across humanitarian, development, climate adaptation, and peacebuilding agendas.

As a conceptual and policy-oriented narrative synthesis integrating peer-reviewed and grey literature, the framework does not seek to establish causal attribution between climate change, migration, and health outcomes. Instead, it supports anticipatory, people-centered responses in contexts where risks are overlapping, dynamic, and shaped by environmental, socioeconomic, and political drivers. The analysis acknowledges uneven evidence, definitional variability, and the non-systematic nature of the review, underscoring the need for comparative country studies, improved indicators, and longitudinal data—both within MENA and in other settings experiencing climate-related mobility under conditions of instability or rapid transformation.

The framework aligns with existing global commitments, including the Sustainable Development Goals, the *Global Compact for Migration*, the *Global Compact on Refugees*, the WHO *Global Action Plan for Refugee and Migrant Health*, and climate policy processes under the UNFCCC. Its contribution lies in enhancing coherence across these agendas while remaining adaptable to different institutional capacities and political contexts. Implementation will involve trade-offs, resource constraints, coordination challenges, and, in some settings, security constraints, reinforcing the importance of leadership, cross-sector governance, partnership, and sustained investment in inclusive and resilient systems.

Forthcoming policy processes—such as the *International Migration Review Forum* in 2026—future climate negotiations, and discussions on the post-2030 development agenda—offer opportunities to further institutionalize integrated climate–migration–health approaches. In this context, greater recognition and operationalization of the Climate–Migration–Health nexus could be considered within the implementation of the *Global Compact for Migration* (GCM), particularly under Objective 7 on reducing vulnerabilities in migration, as well as in future *Sustainable Development Goals* related to Climate Action. Against this backdrop, the NAF-CMH can serve as a practical reference to support policy coherence, strengthen partnerships, and guide incremental implementation. By promoting coordinated, evidence-informed action—while recognizing uncertainty and contextual diversity—the framework aims to contribute to efforts to reduce vulnerability, protect health, and strengthen resilience under escalating climate pressures, within MENA and beyond.

### Limitations

This study is based on a non-systematic narrative review of English-language peer-reviewed and selected grey literature. It does not aim to provide an exhaustive or systematically reproducible synthesis of the evidence, nor to quantify causal relationships between climate change, migration, and health outcomes. Source selection was guided by relevance to policy and practice rather than by formal systematic review protocols, which limits replicability and comprehensive coverage. The inclusion of the grey literature was necessary to capture evolving policy frameworks and operational experience but may reflect institutional priorities and normative positioning. The objective was to synthesize policy-relevant insights across disciplines to inform integrated action.

Empirical evidence on the climate–migration–health nexus—particularly from the MENA region—remains uneven. Evidence from conflict-affected and politically fragile settings is especially constrained by access limitations, data fragmentation, and reporting gaps. Important dimensions, including mental health stressors, gender-based violence, technological innovation, and intersecting vulnerabilities related to age, disability, and socioeconomic status, remain insufficiently documented in the available literature. Variability in terminology and definitions across climate, migration, and health domains further complicates comparability and limits the precision of cross-sector synthesis. These gaps constrain the depth of analysis possible within a conceptual review and highlight priorities for future research.

The Nexus Action Framework (NAF-CMH) is proposed as a policy-oriented and integrative tool derived from existing evidence and expert knowledge. Its components have not been empirically tested as an integrated model, and their operational feasibility across diverse national, institutional, and conflict-affected contexts requires further validation through applied research, country-level studies, and implementation experience. The paper therefore prioritizes strategic framing and policy coherence while recognizing the need for continued empirical refinement and context-specific assessment.

## Figures and Tables

**Table 1:** Climate Change, Migration, and Health: Key Risks in the MENA Region and beyond.

Climate- and Mobility-Related Driver	Key Health Impacts	Populations and Systems Most Affected	Case/Regional Examples
Extreme weather events (heatwaves, floods, storms, wildfires)	Heat-related illness; dehydration; cardiovascular, respiratory, and kidney disease; injuries and fatalities; respiratory harm from wildfire smoke	Outdoor workers (including low-skilled migrant labourers); urban populations; overstretched emergency and primary care services	Bahrain (Al-Sayyad et al., 2014) [30], Kuwait (Alahmad et al., 2022) [31], Oman and Qatar (Umar T, Egbu C, 2018) [32], Qatar (Pradhan et al., 2019) [33], Saudi Arabia (Al-Bouwarthan et al., 2019) [34] (2020) [35], UAE (Aryal et al. 2021) [36]
Air pollution and dust exposure	Worsening asthma, COPD, cardiovascular morbidity and mortality	Migrant workers and urban residents exposed to heat, dust, and poor housing conditions	Kuwait (Alahmad et al., 2023) [31], Sfax/Tunisia (WHO 2016) [37]
Climate-sensitive infectious diseases (vector- and water-borne)	Increased risk of dengue, malaria, cholera, leishmaniasis, schistosomiasis; potential re-emergence of controlled diseases	Displaced populations; communities facing water insecurity, poor sanitation, and environmental degradation	Oman (Al Awaidy, Khamis, 2019) [38], Lebanon (Berjaoui, Al Akoum et al., 2023) [39], MENA Region (Shadomy, Ebrahim, Alii 2025) [40] (Al Salihi, Younise et al., 2024) [41]
Water scarcity and food insecurity	Undernutrition; anaemia; dehydration; diarrhoeal disease	Children, pregnant women, displaced populations, and communities dependent on climate-sensitive livelihoods	MENA Region (FAO 2021) [42]
Mental health impacts	Anxiety, depression, post-traumatic stress disorder, and psychological distress linked to displacement, legal status, loss of livelihoods, and climate uncertainty	Migrants, refugees, internally displaced populations, and informal urban settlements	Documented in displaced and migration-affected communities (Stone et al. 2022 [43]; Anantapong et al., 2024) [44]
Reproductive Health and Gender-Based Violence (GBV):	Limited access to health services, increased maternal mortality, unintended pregnancies and early marriage, sexual exploitation, and other forms of abuse, including human trafficking	Migrant women and girls,	MENA-wide; documented in displaced and migration-affected communities (IOM 2024) [45], Egypt (EcoConServ 2023) [46]
Displacement and migration-related conditions	Exposure to overcrowding, unsafe work environments, limited access to healthcare, and adverse social determinants of health	Migrants, refugees, internally displaced populations, and informal urban settlements	Yemen, Sudan, Iraq, Syria, Jordan (IOM 2023 [3]; Trummer, Mosca et al., 2023 [47])
Health system strain and service disruption	Reduced capacity for routine and emergency care; damage to infrastructure; workforce shortages	Health systems in fragile, conflict-affected, and rapidly urbanising settings; host communities facing increased service demand	Health system responsiveness to refugees and migrants (WHO 2021) [48]
Social tensions linked to service access	Heightened perceptions of competition over housing, water, employment, and health services; risks to social cohesion	Host communities and mobile populations in resource-constrained settings	Urban areas with large migrant inflows, e.g., Lebanese cities (Fakhry et al. 2024) [49] (Henderson et al., 2014) [26]; IOM, 2023 [3]; (Kim K., Ferré Garcia T. 2023) [28].

**Table 2:** Key Components of a Climate-Resilient and Migration-Sensitive Health System: A Building Blocks Approach.

WHO Framework for Health System Strengthening	WHO Guidance on Climate-Resilient Health Systems	Climate Resilient & Migration Sensitive Health Systems
Leadership & Governance	Leadership & Governance	Leadership, Governance & Intersectoral Policy Coherence
Health Workforce	Health Workforce	Climate- and Mobility-Responsive Health Workforce
Health Information System	Vulnerability, Capacity, & Adaptation Assessment	Human Mobility, Climate Vulnerability & Adaptive Capacity Assessment
	Integrated Monitoring & Early Warning	Migration-Sensitive Monitoring, Disaggregated Data & Integrated Information Systems
	Health & Climate Research	Evidence and Research on Climate Change, Human Mobility & Health Intersections
Essential Medical Products & Technology	Climate Resilient & Sustainable Technologies and Infrastructures	Inclusive, Adaptive & Migration-Sensitive Health Technologies and Infrastructures
Service Delivery	Management of Environmental Determinants of Health	Environmental, Social & Structural Determinants of Health for Mobile Populations
	Climate-informed Health Programmes	Migrant-Inclusive Universal Health Coverage & Mobility-Responsive Service Delivery
	Emergency Preparedness & Management	Emergency Preparedness & Crisis Response for Mobile and Displaced Populations
Health Financing	Climate & Health Financing	Equitable, Sustainable, Climate-& Mobility-Responsive Health Financing

**Table 3:** NAF-CMH Climate-Resilient and Migration-Sensitive Health System: Blocks’ Description.

#	Climate-Resilient & Migration-Sensitive Health Systems— (Proposed Building Block)	Explanatory Description
**1**	Leadership, Governance & Intersectoral Policy Coherence	Strategic stewardship within the health system to guide planning, resource allocation, and accountability, ensuring services are responsive to climate- and mobility-related risks. Promotes alignment with relevant sectors—through whole-of-government approaches at national and local levels—so that health system priorities support broader development and governance objectives, enabling integrated, equitable, and context-sensitive responses.
**2**	Climate- and Mobility-Responsive Health Workforce	A health workforce with competencies, planning, and surge capacity aligned to climate- and mobility-related health needs, capable of maintaining essential services and supporting adaptive responses in diverse, high-risk contexts.
**3**	Human Mobility, Climate Vulnerability & Adaptive Capacity Assessment	Integrated health information systems that assess population movements, climate-related health risks, and adaptive capacity to guide targeted interventions and adaptation planning.
**4**	Migration-Sensitive Monitoring, Disaggregated Data & Integrated Information Systems	Health information systems that continuously track health outcomes and the performance of services over time, integrating disaggregated data by population groups and ensuring interoperability with other relevant systems. This supports ongoing surveillance, early warning, and operational decision-making in climate- and mobility-affected contexts.
**5**	Evidence and Research on Climate Change, Human Mobility & Health Intersections	Systematic generation and synthesis of empirical evidence linking climate, mobility, and health to inform policy, planning, and long-term health system strengthening.
**6**	Inclusive, Adaptive & Migration-Sensitive Health Technologies and Infrastructures	Health system–integrated technologies, medical products, and infrastructure designed to be portable, accessible, and adaptable, supporting continuity of care and equitable service delivery for populations affected by climate and mobility.
**7**	Environmental, Social & Structural Determinants of Health for Mobile Populations	Addressing social, environmental, and structural factors shaping health risks and outcomes through communication, health promotion, intersystem coordination, and community engagement for mobile, displaced, and climate-affected populations.
**8**	Migrant-Inclusive Universal Health Coverage & Mobility-Responsive Service Delivery	Ensuring equitable access to essential health services via flexible, adaptive delivery models, continuity of care mechanisms, and integrated service planning that respond to the needs of mobile, displaced, and climate-affected populations.
**9**	Emergency Preparedness & Crisis Response for Mobile and Displaced Populations	Planning, readiness, and rapid-response mechanisms to protect health during shocks, crises, and climate-related events, integrating early warning systems, surge capacity, and cross-sector coordination for mobile, displaced, and climate-affected populations.
**10**	Equitable, Sustainable, Climate- and Mobility-Responsive Health Financing	Financing mechanisms that support equitable access, strengthen long-term system resilience, and enable flexible, adaptive allocation of resources to respond to climate- and mobility-related health challenges, including through cross-sector coordination and integration with broader development and humanitarian funding streams.

## Data Availability

All data used in this study are from published sources, fully referenced in the manuscript. No original datasets were created.

## References

[1] World Bank Group (2021). Middle East & North Africa. Climate Roadmap. Driving Transformational Climate Action and Green Recovery in MENA. 2021–2025.

[2] World Bank Group (2023). Climate Change Action in the Middle East and North Africa—Key Insights from Country Climate and Development Reports.

[3] International Organization for Migration (IOM) (2023). Region on the Move: Regional Mobility Report for the Middle East and North Africa 2021–2022.

[4] WHO (2023). Climate Change, Health and Environment: A Regional Framework for Action, 2023–2029.

[5] Borghesi S., Ticci E. (2019). Climate Change in the MENA Region: Environmental Risks, Socioeconomic Effects and Policy Challenges for the Future.

[6] International Organization for Migration (IOM) (2024). Sustainable Cities, Thriving Migrants: Enhancing Urban Livability for Migrants amidst Climate Challenges in MENA Cities.

[7] Mosca D.T., Vearey J., Orcutt M., Zwi A.B. (2020). Universal Health Coverage: Ensuring migrants and migration are included. Glob. Soc. Policy.

[8] Lee H., Romero J., IPCC (2023). 2023: Summary for Policymakers. Climate Change 2023: Synthesis Report.

[9] Schewel K. (2020). Understanding Immobility: Moving Beyond the Mobility Bias in Migration Studies. Int. Migr. Rev..

[10] McMichael C. (2020). Human mobility, climate change, and health: Unpacking the connections. Lancet Planet. Health.

[11] Silenzi A., Marotta C., Caredda E., Machado R.S., Severoni S., Rezza G. (2023). Climate change, human migration and health nexus: What do we know about public health implications on a global scale. Epidemiol. Prev..

[12] WHO (2015). Operational Framework for Building Climate-Resilient Health Systems.

[13] Diab J.L., Scissa C. (2023). Gender, Migration and Environment in the MENA: Vulnerabilities, Frameworks and Ways Forward. J. Migr. Aff..

[14] Diab J.L. (2024). Chapter 8: Refuge in silos: How three of the largest MENA hosts address Syrian mass displacement in the absence of coordinated efforts and responses. Research Handbook on Asylum and Refugee Policy.

[15] International Organization for Migration (IOM) (2025). Region on the Move: Regional Mobility Report for the Middle East and North Africa 2025.

[16] Zaccara L., dos Santos Gonςalves M. (2021). Migrants, Refugees, and Displaced Persons in the Middle East and North Africa: An approach from the Global South. REMHU Rev. Interdiscip. Mobil. Hum..

[17] Miniaoui H., Ouni H. (2019). Economy & Territory. Workers’ Remittances and Economic Growth in MENA Countries: The Role of Financial Development. Mediterranean Yearbook 2020.

[18] International Labour Office (2023). Assessment of Labour Migration Statistics in the Arab States: Regional Report.

[19] International Organization for Migration, Global Data Institute (2023). Regional Snapshot: The Middle East & North Africa—Quarterly Report (April–June 2023); Displacement Tracking Matrix.

[20] IDMC (2022). Contribution of the Internal Displacement Monitoring Centre (IDMC) to the Special Rapporteur on the Human Rights of Internally Displaced Persons’ HRC56 Thematic Report on Climate Change and Internal Displacement.

[21] Beyer R., Milan A. (2023). Climate Change and Human Mobility: Quantitative Evidence on Global Historical Trends and Future Projections.

[22] Cárdenas-Vélez M., Barrott J., Betancur Jaramillo J.C., Hernández-Orozco E., Maestre-Másmela D., Lobos-Alva I. (2024). A combined cognitive and spatial model to map and understand climate-induced migration. Environ. Dev. Sustain..

[23] Mamshai F.H.A. (2023). “Climate Change as a Threat Multiplier”: Security and Communal Implications for Iraq. Community Change.

[24] International Organization for Migration (2023). Climate Action Overview Middle East and North Africa.

[25] Boehm S., Schumer C. (2023). 10 Big Findings from the 2023 IPCC Report on Climate Change.

[26] Henderson J.V., Storeygard A., Deichmann U. (2014). 50 Years of Urbanization in Africa: Examining the Role of Climate Change.

[27] International Organization for Migration, Regional Office for Middle East and North Africa (2023). Climate Change, Conflict, Migration (CCM); Desk Review.

[28] Kim K., Ferré Garcia T. (2023). Climate Change and Violent Conflict in the Middle East and North Africa. Int. Stud. Rev..

[29] UNHCR (2024). Climate Change and Displacement.

[30] Al-Sayyad A.S., Hamadeh R.R. (2014). The Burden of climate-related conditions among laborers at Al-Razi Health Centre, Bahrain. J. Bahrain Med. Soc..

[31] Alahmad B., Vicedo-Cabrera A.M., Chen K., Garshick E., Bernstein A.S., Schwartz J., Koutrakis P. (2022). Climate change and health in Kuwait: Temperature and mortality projections under different climatic scenarios. Environ. Res. Lett..

[32] Umar T., Egbu C. (2018). Heat Stress, a Hidden Cause of Accidents in Construction. ICE Proc. Munic. Eng..

[33] Pradhan B., Kjellstrom T., Atar D., Sharma P., Kayastha B., Bhandari G., Pradhan P.K. (2019). Heat stress impacts on cardiac mortality in Nepali migrant workers in Qatar. Cardiology.

[34] Al-Bouwarthan M., Quinn M.M., Kriebel D., Wegman D.H. (2019). Assessment of heat stress exposure among construction workers in the hot desert climate of Saudi Arabia. Ann. Work. Expo. Health.

[35] Al-Bouwarthan M., Quinn M.M., Kriebel D., Wegman D.H. (2020). Risk of Kidney Injury among Construction Workers Exposed to Heat Stress: A Longitudinal Study from Saudi Arabia. Int. J. Environ. Res. Public Health.

[36] Aryal N., Regmi P.R., Sedhain A., Krishna KC R., Martinez Faller E., Rijal A., van Teijlingen E. (2021). Kidney health risk of migrant workers: An issue we can no longer overlook. Health Prospect.

[37] WHO (2016). Health and Climate Change: Country Profile: Tunisia.

[38] Al Awaidy S.T., Khamis F. (2019). Dengue fever: An emerging disease in Oman requiring urgent public health interventions. Oman Med. J..

[39] Berjaoui C., Al Akoum N., El Nouiri A., Khayat S., Abbass M., Al Mousawi A., Wellington J., Uwishema O. (2023). A minireview of cholera outbreak in Lebanon—A rising public health concern. Ann. Med. Surg..

[40] Shadomy S.V., Ebrahim S.H., Guagliardo S.A.J., Sánchez-González L., Zureick K., Sinclair J.R., A Schneider D., Walker A.T., Payne D.C., Vieira A.R. (2025). Emerging and re-emerging disease threats in the Middle East and North Africa region—One Health approaches and potential strategies. Eur. J. Public Health.

[41] Al Salihi K.A., Younise M.H., Mahmoud Z.Z., Hussain T. (2024). The 2022 Crimean–Congo hemorrhagic fever. Austral J. Vet. Sci..

[42] FAO, IFAD, UNICEF, WFP, WHO (2021). The State of Food Security and Nutrition in the World 2021.

[43] Stone K., Blinn N., Spencer R. (2022). Mental health impacts of climate change on women: A scoping review. Curr. Environ. Health Rep..

[44] Anantapong K., Moura H.F., Udomratn P., Persaud A., Javed A., Ramachandran P., Castaldelli-Maia J.M., Torales J., Ventriglio A., Bhugra D. (2024). Geopsychiatry: Climate change, migration, mental health. Ind. Psychiatry J..

[45] International Organization for Migration (2024). Effects of Climate Mobility on Women and Girls in the MEAN Region.

[46] EcoConServ (2023). Assessing the Impact of Climate Change on Women and Girls’ Reproductive Health in Egypt.

[47] Trummer U., Ali T., Mosca D., Mukuruva B., Mwenyango H., Novak-Zezula S. (2023). Climate change aggravating migration and health issues in the African context: The views and direct experiences of a community of interest in the field. J. Migr. Health.

[48] WHO (2021). Mapping Health Systems’ Responsiveness to Refugee and Migrant Health Needs.

[49] Fakhry Y., Hassan H.F., Diab J.L. (2024). Health in Crisis: A Paradox of Access for Syrian Refugees and Lebanese Hosts. Health Serv. Insights.

[50] Al Thani S.M.B.H. The role of Public Health in Heat stress and related illnesses. Proceedings of the International Conference on Occupational Heat Stress: “Implementation of Practices, Sharing of Experiences”.

[51] Alshehari A.H., Al-Selwi A.A., Agu S.A., Younes M.A. (2024). Measuring progress towards universal health coverage in 22 Middle East and North African countries. Dialog. Health.

[52] Lasater M.E., Prager G., Choi Y.A., Groteclaes T., Rao D., Kamps S.P., Altare C., Spiegel P.B. (2025). Understanding relationships among climate change, conflict, migration/displacement and health in humanitarian settings: A scoping review. Confl. Health.

[53] Mena R., Brown S., Peters L.E., Kelman I., Ji H. (2022). Connecting Disasters and Climate Change to the Humanitarian-Development-Peace Nexus. J. Peacebuilding Dev..

[54] Jackson P. (2007). From Stockholm to Kyoto: A Brief History of Climate Change.

[55] Stockholm Declaration. UN Doc. A/CONF.48/14, at 2 and Corr.1. Proceedings of the United Nations Conference on the Human Environment.

[56] WMO/UNEP Report of the First Session of the Intergovernmental Panel on Climate Change. IPCC-1. Geneva 9–11 November 1988. World Climate Programme Publications Serie. TD-No 267. https://archive.ipcc.ch/meetings/session01/first-final-report.pdf.

[57] (1992). United Nations Framework Convention on Climate Change. FCCC/INFORMAL/84 GE.05-62220 (E) 200705. https://unfccc.int/resource/docs/convkp/conveng.pdf.

[58] Adger N., Aggarwal P., Agrawala S., Alcamo J., Allali A., Anisimov O., Arnell N., Boko M., Canziani O., WMO/UNEP (2007). Climate Change 2007: Impacts, Adaptation and Vulnerability.

[59] WHO Climate Change and Health. Agenda item 11.11. Proceedings of the Sixty-first World Health Assembly.

[60] WHO Health of migrants. WHA61.17 Agenda item 11.9. Proceedings of the Sixty-first World Health Assembly.

[61] Costello A., Abbas M., Allen A., Ball S., Bell S., Bellamy R., Friel S., Groce N., Johnson A., Kett M. (2009). *Managing the health effects of climate change*: Lancet and University College London Institute for Global Health Commission. Lancet.

[62] International Organization for Migration (2008). Migration and Climate Change.

[63] McMichael C., Barnett J., McMichael A.J. (2012). An Ill Wind? Climate Change, Migration, and Health. Environ. Health Perspect..

[64] UNDP (United Nations Development Program) (2009). Human Development Report 2009. Overcoming Barriers: Human Mobility and Development.

[65] WHO, IOM (2010). Health of Migrants—The Way Forward Report of a Global Consultation.

[66] Pachauri R.K., Meyer L.A., IPCC, Core Writing Team (2014). Climate Change 2014: Synthesis Report. Contribution of Working Groups I, II and III to the Fifth Assessment Report of the Intergovernmental Panel on Climate Change.

[67] WHO Second Global Conference on Health and Climate Change. Conference Conclusions and Action Agenda. Paris 2016. https://2022.neha.org/sites/default/files/eh-topics/climate-change/Second-Global-Conference-Health-Climate-Conclusions-2016.pdf.

[68] UNGA Resolution adopted by the General Assembly: New York Declaration for Refugees and Migrants. Proceedings of the Seventy-First Session of the United Nations General Assembly.

[69] Puebla Fortier J., Mosca D.T., Weekers J., Barragan E., Gushulak B., Ingleby D., Siriwardhana C., Roberts B., McKee M., Huddleston T. (2017). Health of Migrants: Resetting the Agenda.

[70] Abubakar I., Aldridge R.W., Devakumar D., Orcutt M., Burns R., Barreto M.L., Dhavan P., Fouad F.M., Groce N., Guo Y. (2018). The UCL–Lancet Commission on Migration and Health: The health of a world on the move. Lancet.

[71] Hunter L., Henry S., McMichael C., Bocquier P. (2018). Climate, Migration and Health: An Underexplored Intersection.

[72] Schütte S., Gemenne F., Zaman M., Flahault A., Depoux A. (2018). Connecting planetary health, climate change, and migration. Lancet Planet.-Health.

[73] Schwerdtle P., Bowen K., McMichael C. (2018). The health impacts of climate-related migration. BMC Med..

[74] Villa S., Raviglione M.C. (2019). Migrants’ health: Building migrant-sensitive health systems. J. Public Health Res..

[75] Schwerdtle P.N., McMichael C., Mank I., Sauerborn R., Danquah I., Bowen K.J. (2020). Health and migration in the context of a changing climate: A systematic literature assessment. Environ. Res. Lett..

[76] Nayna Schwerdtle P., Stockemer J., Bowen K.J., Sauerborn R., McMichael C., Danquah I. (2020). A Meta-Synthesis of Policy Recommendations Regarding Human Mobility in the Context of Climate Change. Int. J. Environ. Res. Public Health.

[77] WHO (2022). Policy Brief. Loss and Damage.

[78] IOM, WHO, Lancet-Migration (2021). The Climate Change, Health and Migration Nexus at the 2021 UN Climate Conference (COP26, Oct–Nov 2021), Glasgow, United Kingdom.

[79] WHO (2021). Alliance for Transformative Action on Climate-and-Health. https://www.who.int/initiatives/alliance-for-transformative-action-on-climate-and-health.

[80] WHO COP26—Direct Linkages Between Climate Change, Health and Migration Must Be Tackled Urgently—IOM, WHO, Lancet-Migration. https://www.who.int/news/item/09-11-2021-cop26---direct-linkages-between-climate-change-health-and-migration-must-be-tackled-urgently-iom-who-lancet-migration.

[81] UNHCR The Climate Resilience Fund. https://www.unhcr.org/sites/default/files/2025-06/factsheet-climate-resilience-fund.pdf.

[82] Mayors Migration Council (MMC) Global Cities Fund for Migrants and Refugees. https://mayorsmigrationcouncil.org/gcf/.

[83] Lancet-Migration European Regional Hub (2021). Climate Change, Migration, and Health Research in the European Region.

[84] Reuveny R. (2021). Climate-related migration and population health: Social science-oriented dynamic simulation model. BMC Public Health.

[85] McMichael C. (2023). Climatic and Environmental Change, Migration, and Health. Annu. Rev. Public Health.

[86] Messeri A., Morabito M., Bonafede M., Bugani M., Levi M., Baldasseroni A., Binazzi A., Gozzini B., Orlandini S., Nybo L. (2019). Heat stress perception among native and migrant workers in Italian industries—Case studies from the construction and agricultural sectors. Int. J. Environ. Res. Public Health.

[87] Romanello M., McGushin A., Di Napoli C., Drummond P., Hughes N., Jamart L. (2021). The 2021 report of the Lancet Countdown on health and climate change: Code red for a healthy future. Lancet.

[88] Tenzing J.D. (2020). Integrating social protection and climate change adaptation: A review. Wiley Interdisc. Rev. Clim. Change.

[89] Barnett J. (2022). Global environmental change III: Political economies of adaptation to climate change. Prog. Hum. Geogr..

[90] UNNM (2022). Act Now: Migrant Inclusion in Climate Action Is an Obligation, Not an Option.

[91] UNFCCC (COP 28) Guiding Principles for Financing Climate and Health Solutions. Proceedings of the World Climate Action Summit.

[92] Romanello M., Walawender M., Hsu S.-C., Moskeland A., Palmeiro-Silva Y., Scamman D., Smallcombe J.W., Abdullah S., Ades M., Al-Maruf A. (2025). The 2025 report of the Lancet Countdown on health and climate change: Climate change action offers a lifeline. Lancet.

[93] Green Climate Fund (2024). Bridging the Climate-Health Gap. https://www.greenclimate.fund/insights/bridging-climate-health-gap.

[94] World Health Organization (2025). Delivering the Belém Health Action Plan: COP30 Special Report on Health and Climate Change.

[95] Platform on Disaster Displacement (2024). Perspective. COP29 Key Messages. https://disasterdisplacement.org/perspectives/cop29-key-messages/.

[96] Issa R., Sarsour A., Cullip T., Toma S., Ruyssen I., Scheerens C. (2023). Gaps and opportunities in the climate change, migration and health nexus: Insights from a questionnaire based study of practitioners and researchers. J. Migr. Health.

[97] Khalid A., Babry J.A., Vearey J., Zenner D. (2023). Turning up the heat: A conceptual model for understanding the migration and health in the context of global climate change. J. Migr. Health.

[98] Bharadwaj R., Huq S. (2022). Climate-induced migration and health issues: A toolkit for policymakers.

[99] Schwerdtle N., Devine C., Berner-Rodoreda A., McMahon S., Barnighausen K. (2024). Adapting to Climate Change: Strategies and Perspectives from Humanitarian Health Workers—A Qualitative Study. J. Clim. Change Health.

[100] Pörtner H.O., Roberts D.C., Poloczanska E.S., Mintenbeck K., Tignor M., Alegría A., Craig M., Langsdorf S., Löschke S., Möller V. (2022). IPCC, 2022: Summary for policymakers. Climate Change 2022: Impacts, Adaptation, and Vulnerability.

[101] Severoni S., Hiam L., Garry S. (2024). Climate Change and Health: Displaced and Migrant Populations Must Be Included. Lancet.

[102] WHO (2023). Global Research Agenda on Health, Migration and Displacement: Strengthening Research and Translating Research Priorities into Policy and Practice.

[103] WHO EMRO (2023). Second High-Level Interregional Meeting on the Health of Refugees and Migrants, SharmEl Sheikh, Egypt, 16–17 March 2023.

[104] WHO (2025). Health System Strengthening Interventions to Improve the Health of Displaced and Migrant Populations in the Context of Climate Change.

[105] International Organization for Migration (IOM) (2025). Positioning the Climate Change–Health–Migration Nexus in the Middle East and North Africa Region: A Framework for Policy and Action.

[106] WHO, IOM, UNHCR, Government of Morocco Rabat Declaration. Proceedings of the High-level segment of the 3rd Global Consultation on the Health of Refugees and Migrants.

[107] International Organization for Migration, World Health Organization, United Nations Office for Disaster Risk Reduction (2024). IOM, WHO and UNDRR Launch Regional Health Programme Addressing Climate Change Impacts in Jordan, Iraq and Lebanon. https://www.undrr.org/news/un-agencies-launch-joint-programme-support-iraq-jordan-and-lebanon-climate-change-response.

[108] Figueiredo S., Dierks A., Ferreira R. (2024). Mental health screening in refugee communities: Ukrainian refugees and their post-traumatic stress disorder specificities. Eur. J. Trauma Dissociation.

[109] Jordan Ministry of Health (2025). Jordan’s National Health Sector Climate Adaptation Strategy 2024–2033.

[110] World Health Organization Regional Office for the Eastern Mediterranean (2025). Climate Change and Health in the Eastern Mediterranean Region: From Framework to Action. https://www.emro.who.int/media/news/climate-change-and-health-in-the-eastern-mediterranean-region-from-framework-to-action.html.

[111] Ministry of Public Health (Lebanon) (2023). Lebanon Health Strategy: Vision 2030. https://www.moph.gov.lb/en/view/67044/lebanon-national-health-strategy-vision-2030.

[112] UNICEF & WHO (2021). Progress on Household Drinking Water, Sanitation and Hygiene 2000–2020: Five Years into the SDGs. https://www.who.int/publications/i/item/9789240030848.

[113] UNHCR & UNDP (2024). Regional Refugee and Resilience Plan (3RP): Regional Strategic Overview 2025. https://www.3rpsyriacrisis.org/wp-content/uploads/2024/12/3RP_Regional_Strategic_Overview_2025.pdf.

[114] Human Rights Watch (2024). Gulf States: Inadequate Heat Protection Putting Workers in Peril. https://www.hrw.org/news/2024/08/08/gulf-states-inadequate-heat-protection-putting-workers-peril.

[115] International Labour Organization (2023). Advancing Social Protection for Migrant Workers in the GCC Countries: New Ambition Emerges from Discussion at the Global Forum for Migration and Development Summit. https://www.ilo.org/resource/news/advancing-social-protection-migrant-workers-gcc-countries-new-ambition.

[116] Lyles E., Hanquart B., Woodman M., Doocy S., The LHAS Study Team (2016). Health service utilization and access to medicines among Syrian refugee and host community children in Lebanon. J. Int. Humanit. Action.

[117] Sethi S., Jonsson R., Skaff R., Tyler F. (2017). Community-based noncommunicable disease care for Syrian refugees in Lebanon. Glob. Health Sci. Pract..

[118] Nakkash R., Fares M., Tleis M., Mugharbil S., Antaby M., Al Masri H., Ghandour L., Al Halabi F., Najjar Y., Louis B. (2024). Power sharing in community-engaged research with Syrian refugees in Lebanon: Using community engagement to shape intervention fit to context. SSM Ment. Health.

[119] GIZ Improving Water Security for Displaced People in Jordan: Supporting Participatory Resource Management to Stabilize the Situation in Host Communities. https://www.giz.de/en/projects/supporting-participatory-resource-management-stabilize-situation-host-communities?utm_source=chatgpt.com.

[120] Evangelidou S., Seedat F., Deal A., Ouahchi A., Maatoug T., Elafef E., Edries H., Bouaddi O., Abdellatifi M., Arias S. (2025). Migrant Health Country Profile tool (MHCP-t) for transforming health data collection and surveillance in the Middle East and North African (MENA) region: Tool development protocol with embedded process evaluation. BMJ Open.

[121] World Health Organization Regional Office for the Eastern Mediterranean (2024). Achieving integrated disease surveillance in the Eastern Mediterranean Region. East. Mediterr. Health J..

[122] IDRC-International Development Research Centre (2025). MENA Hub for Action Research on Climate Change and Health. https://idrc-crdi.ca/en/what-we-do/projects-we-support/project/mena-hub-action-research-climate-change-and-health.

[123] Nash S., van der Voort E.C.A. (2024). Green Futures: The Role of Labour Migration in Advancing Climate Action.

[124] International Labour Organization (2019). THAMM Plus Programme: Towards a Holistic Approach to Labour Migration Governance and Labour Mobility in North Africa. 2019–2023. https://www.ilo.org/projects-and-partnerships/projects/towards-holistic-approach-labour-migration-governance-and-labour-mobility-0.

[125] International Labour Organization (2023). Sponsorship Reform and Internal Labour Market Mobility for Migrant Workers in the Arab States. https://www.ilo.org/resource/other/sponsorship-reform-and-internal-labour-market-mobility-migrant-workers-arab.

[126] (2023). Migrant-Rights.Org Comparison of Health Care Coverage for Migrant Workers in the GCC. https://www.migrant-rights.org/2023/09/comparison-of-health-care-coverage-for-migrant-workers-in-the-gcc/.

[127] World Bank Group Middle East & North Africa Climate Road Map 2021–2025. https://www.worldbank.org/en/region/mena/publication/middle-east-north-africa-climate-roadmap.

[128] Watson C., Schalatek L., Evéquoz A. (2025). Climate Finance Regional Briefing: Middle East and North Africa. Climate Funds Update. ODI Global. https://climatefundsupdate.org/wp-content/uploads/2025/03/CFF9-2025-ENG-MENA-DIGITAL.pdf?utm_source=chatgpt.com.

